# FMRP modulates the Wnt signalling pathway in glioblastoma

**DOI:** 10.1038/s41419-022-05019-w

**Published:** 2022-08-18

**Authors:** Giorgia Pedini, Mariachiara Buccarelli, Fabrizio Bianchi, Laura Pacini, Giulia Cencelli, Quintino Giorgio D’Alessandris, Maurizio Martini, Stefano Giannetti, Franceschina Sasso, Valentina Melocchi, Maria Giulia Farace, Tilmann Achsel, Luigi M. Larocca, Lucia Ricci-Vitiani, Roberto Pallini, Claudia Bagni

**Affiliations:** 1 Department of Biomedicine and Prevention, University of Rome Tor Vergata, Rome, Italy; 2 Department of Oncology and Molecular Medicine, Istituto Superiore di Sanità, Rome, Italy; 3 Fondazione IRCCS Casa Sollievo della Sofferenza, Unit of Cancer Biomarkers, San Giovanni Rotondo, Italy; 4 UniCamillus, Saint Camillus International University of Health and Medical Sciences, Rome, Italy; 5 Institute of Neurosurgery, Fondazione Policlinico Universitario A. Gemelli IRCCS - Università Cattolica del Sacro Cuore, Rome, Italy; 6 Department of Human & Childhood Pathology “G. Barresi”, University of Messina School of Medicine, Messina, Italy; 7 Department of Neuroscience, Institute of Anatomy, Università Cattolica del Sacro Cuore, Rome, Italy; 8 Department of Fundamental Neurosciences (DNF), University of Lausanne, Lausanne, Switzerland; 9 Pathology, Fondazione Policlinico Universitario A. Gemelli IRCCS - Università Cattolica del Sacro Cuore, Rome, Italy

**Keywords:** Cancer stem cells, Developmental disorders

## Abstract

Converging evidence indicates that the Fragile X Messenger Ribonucleoprotein (FMRP), which absent or mutated in Fragile X Syndrome (FXS), plays a role in many types of cancers. However, while FMRP roles in brain development and function have been extensively studied, its involvement in the biology of brain tumors remains largely unexplored. Here we show, in human glioblastoma (GBM) biopsies, that increased expression of FMRP directly correlates with a worse patient outcome. In contrast, reductions in FMRP correlate with a diminished tumor growth and proliferation of human GBM stem-like cells (GSCs) in vitro in a cell culture model and in vivo in mouse brain GSC xenografts. Consistently, increased FMRP levels promote GSC proliferation. To characterize the mechanism(s) by which FMRP regulates GSC proliferation, we performed GSC transcriptome analyses in GSCs expressing high levels of FMRP, and in these GSCs after knockdown of FMRP. We show that the WNT signalling is the most significantly enriched among the published FMRP target genes and genes involved in ASD. Consistently, we find that reductions in FMRP downregulate both the canonical WNT/β-Catenin and the non-canonical WNT-ERK1/2 signalling pathways, reducing the stability of several key transcription factors (i.e. β-Catenin, CREB and ETS1) previously implicated in the modulation of malignant features of glioma cells. Our findings support a key role for FMRP in GBM cancer progression, acting via regulation of WNT signalling.

## Introduction

Glioblastoma (GBM), the most frequent and malignant primary brain tumor in adults [1], is characterized by uncontrolled cellular proliferation, robust angiogenesis, propensity for necrosis, diffuse infiltration, resistance to apoptosis and genomic instability [2]. Even with current multimodal therapies, including maximal safe resection and radiotherapy supported by treatment with the alkylating agent temozolomide (TMZ), the mean survival rate for GBM patients is only 15 months [3]. Over the last decades, comprehensive studies have revealed a profound cellular and molecular heterogeneity in GBM [4–8]. The identification of a small population of tumor-initiating cells with stem properties, termed GBM stem-like cells (GSCs), in perivascular and hypoxic niches introduced a new and revolutionary target for therapy. GSCs, uniquely endowed with self-renewal capacity, multi-potency and induction of tumorigenesis [9, 10], are considered the primary cause of GBM chemo/radio-resistance and relapse, representing a relevant target for medical interventions [11–13].

The intricate molecular network underlying tumor progression makes highly complex the identification of potential pharmacological treatments. RNA-binding proteins (RBPs) are emerging as key regulators in cancer development, playing a key role in post-transcriptional control and gene expression homeostasis [14]. In this scenario, growing evidence shows the overexpression of the RBP Fragile X Mental Retardation Protein (FMRP) in several kinds of tumors, regulating cancer progression and invasiveness [15–18]. The *FMR1* gene, encoding FMRP, is expressed in different tissues and cancer cell types (https://www.genevestigator.com/gv/).

FMRP is an RBP involved in multiple aspects of the mRNA metabolism, including negative regulation of translation [19–23], modulation of mRNA stability [24–26], transport [27–29], pre-mRNA splicing [30, 31] or RNA editing [32–34].

In addition to its well-established functions in the brain, independent clinical and molecular indications suggest that FMRP may also play a role in tumorigenesis. The absence of FMRP is known to cause Fragile X Syndrome (FXS), the most frequent form of inherited intellectual disability and syndromic autism [35]. Interestingly, a decreased risk of tumor incidence has been reported in a Danish cohort of patients with FXS [36]. Molecularly, a large subset of mRNAs, which play major roles in brain function and cancer, are targets of FMRP [37], and several FMRP protein interactors are mutated in various cancers [38]. Clinically, FMRP has been shown to be overexpressed in highly aggressive triple-negative breast cancers (TNBC). High FMRP levels also lead to increased number of metastasis through the regulation of mRNAs encoding proteins involved in epithelial to mesenchymal transition (EMT) [39]. Furthermore, recent data have shown that dysregulation of the *N*-methyl-D-aspartate (NMDA) receptor signalling pathway results in aberrant FMRP levels in pancreatic neuroendocrine tumors [40]. Finally, enhanced FMRP expression promotes proliferation through activation of MEK/ERK signalling in astrocytomas [41]. Notably, a case study of a boy with FXS diagnosed with GBM at 10 years old reported no specific neurological abnormalities throughout the clinical assessment, until the age of 18 [42]. Combined, these findings suggest that the absence of FMRP might have a “protective” effect against tumor growth, including in GBMs.

Here we investigated the contribution of FMRP to the biology of the most aggressive brain cancer, namely GBM. Given the importance of GSCs in GBM progression and their emerging role as therapeutic target, we investigated the molecular mechanisms driving GBM aggressiveness by analyzing GSCs derived from 28 GBM patients. Our data show that FMRP downregulation affects GSC proliferation ability in vivo and in vitro. In addition, transcriptome analysis highlights the WNT mediated signalling as a key dysregulated pathway upon FMRP reduction, with this inhibition of canonical and non-canonical WNT pathway leading to decreased expression and activity of different transcription factors, known to affect GMB proliferation. These findings demonstrate the key role for FMRP in the aggressive proliferation of GSCs and support the hypothesis that FMRP may constitute a potential therapeutic target for GBM.

## Materials and Methods

### Patients, diagnosis, and tumor characterization

Tumor tissue samples were derived from adult patients with GBM tumors (WHO grade IV) undergoing surgical resection at the Institute of Neurosurgery, Catholic University School of Medicine in Rome. Informed consent was obtained from the patients before surgery. All experiments involving human specimens were conformed to the principles described in the NMA Declaration of Helsinki and the NIH Belmont report. The expression of the proliferation marker Ki-67 and phosphatase and tension homolog (PTEN) were characterized on tumor specimen by immunohistochemistry. O6-methylguanine-DNA methyltransferase (MGMT) promoter methylation patterns were assessed on genomic DNA extracted from paraffin-embedded tissue by methylation-specific PCR as previously described [43]. Levels of VEGF and EGFRvIII were assessed as previously described [44, 45].

### Establishing GSC cultures

GSCs from 28 patients were isolated and characterized. Surgical specimens were subjected to mechanical dissociation and the resulting cell suspension was cultured in a serum-free medium supplemented with EGF and FGF as previously described [43]. Cell lines actively proliferating require 3–4 weeks to be established. In these conditions, cells grew as clusters (neurospheres) of undifferentiated cells, as indicated by morphology and expression of stem cell markers such as CD133, SOX2, Musashi-1, and nestin. The in vivo tumorigenic potential of GBM neurospheres was assayed by intracranial or subcutaneous cell injection in immunocompromised mice. GBM neurospheres were able to generate tumors with histological features mirroring the human parent tumor. GSC lines were validated by Short Tandem Repeat (STR) DNA fingerprinting. Nine highly polymorphic STR loci plus amelogenin (Cell IDTM System, Promega Inc., Madison, WI, USA) were used. Detection of amplified fragments was obtained by ABI PRISM 3100 Genetic Analyzer (Applied Biosystems, Carlsbad, CA, USA). Data analysis was performed by GeneMapper^®^ software, version 4.0 (Biological Bank and Cell Factory, National Institute for Cancer Research, IST, Genoa, Italy). All GSC line profiles were challenged against public databases to confirm authenticity.

### Mice and animal care

Immunosuppressed SCID mice used in this study are male, 4–6 weeks old, 20–25 g of body weight (Charles River, Milan, Italy). Mice were kept under pathogen-free conditions in positive-pressure cabinets (Tecniplast Gazzada, Varese, Italy) and observed daily for neurological behavior. A 12-h light/dark cycle was used, and food and water were available ad libitum. Experiments involving animals were approved by the Ethical Committee of the Università Cattolica del Sacro Cuore (UCSC) in Rome (Pr. No. CESA/P/51/2012). Animal experiments described in this study were conducted according to the guidelines set by the European Directive 2010/63/EU and the European Recommendations 526/2007 and the Italian D.Lgs 26/2014. Sample size for animal studies was chosen in order to have the smallest number of cases that allowed a statistical analysis.

### Glioblastoma cell Line

The human GBM cell line T98G was purchased from the American Type Culture Collection (Manassas, VA, USA). The GBM cell line was cultured in Dulbecco’s Modified Eagle’s Medium (DMEM; Thermo Fisher Scientific, MA, USA), supplemented with 10% fetal bovine serum (FBS; Thermo Fisher Scientific, MA, USA) and 1% penicillin/streptomycin (Thermo Fisher Scientific, MA, USA) at 37 °C with 5% CO_2_.

### Protein extract preparation

GSCs were lysed in ice-cold buffer (250 mM NaCl, 10 mM Tris/HCl pH 7.4, 10 mM MgCl2, 1% Triton X-100, 10 µl/ml Protease inhibitor cocktail (Roche, Mannheim, Germany), 10 µl/ml Phosphatase inhibitor cocktails II and III (Merck KGaA, Darmstadt, Germany), 40 U/ml RNaseOUT (Invitrogen, Thermo Fisher Scientific, MA, USA).

### Western blotting

Standard methodologies were used. Protein extracts were separated by SDS–PAGE electrophoresis, and transferred to a PVDF membrane (GE Healthcare, Milan, Italy). FMRP levels were detected using a specific polyclonal antibody, namely PZ1. A synthetic peptide corresponding to aminoacid 548 to 564 of the mouse FMRP (gene ID 14265) was used for the production of the rabbit polyclonal antibodies employed in this study (21st Century Biochemicals). As shown in the Fig. S1A, the antibody recognizes at least three FMRP isoforms in mouse neuronal and brain protein extracts and at least two FMRP isoforms in human fibroblasts and iPSCs protein extracts).

Membranes were incubated using the following specific antibodies including human anti-Vinculin (1:2000, Merck, Cod. V9131), anti-GSK3β (1:1000, Cell Signaling Technology Inc., Danvers, MA, USA, Cod. 4337), anti-phospho-GSK3β (Ser9, 1:1000, Cell Signaling Technology Inc., Cod. 5558), anti-β-Catenin (1:2000, Invitrogen, Cod. 13-8400), anti-WNT5B (1:500, Abcam, Cambridge, MA, USA, ab124818), anti-p44/42 MAPK (Erk1/2, 1:1000, Cell Signaling Technology Inc., Cod. 137F5), anti-phospho-p44/42 MAPK (Erk1/2 Thr202/Tyr204, 1:1000, Cell Signaling Technology Inc., Cod. 9101), anti-ETS1 (1:1000, Cell Signaling Technology Inc., Cod. 14069), anti-phospho-ETS1 (T38, 1:500, Abcam, Cod. ab59179), anti-CREB (1:1000, Cell Signaling Technology Inc., Cod. 48H2), anti-phospho-CREB (Ser133, 1:1000, Cell Signaling Technology Inc., Cod. 87G3), anti-FXR2P (1:1000, Merck, Cod. F1554). Secondary HRP-conjugated anti-rabbit, anti-mouse antibodies (1:10000) were purchased from Promega (Milan, Italy, Cod. W4011 and W402B, respectively). Proteins were revealed using an enhanced chemiluminescence kit (Bio-Rad) and the imaging system LAS-4000 mini (GE Healthcare). Quantification was performed using the IQ ImageQuant TL software (GE Healthcare). Coomassie staining of the membranes and Vinculin signals were used as normalizers.

The full and uncropped figures are provided as Supplemental Material.

### Quantitative real-time-PCR (RT-qPCR)

Total RNA was extracted from GSCs with TRIzol according to the manufacturer’s protocol (Life Technologies, Thermo Fisher Scientific, Cod. 15596026). For the cDNA synthesis 500 ng of total RNA was used as input into a 20 μl reaction using p(dN)6 and 200U/ μl M-MLV RTase (Invitrogen, Thermo Fisher Scientific, 28025013). mRNAs were quantified by real-time PCR using SYBR^®^ Green Master Mix (Bio-Rad, Segrate (MI), Italy) on StepOnePlus™ Real-Time PCR machine (Life Technologies, Thermo Fisher Scientific) according to the manufacturer’s instructions using specific primers. The mRNAs levels were expressed in relative abundance compared to *HPRT1* or *H3* gene (2ˆ(-delta delta CT) method). Specific primers were used for the amplification of the selected genes:

*hFMR1* 5′-TGTCAGATTCCCACCTCCTG-3′*;* 5′-TAACCACCAACAGCAAGCCT-3′

*hHPRT1* 5′-TGCTGAGGATTTGGAAAGGGT-3′*;* 5′-TCGAGCAAGACGTTCAGTCC-3′

*hACTB* 5′-ACCGAGCGCGGCTACAG-3′*;* 5′-CTTAATGTCACGCACGATTTCC-3′

*hWNT5B* 5′-GCAGAAGGTTGACAGCTTCAGT-3′*;* 5′-ACAGTTTCCAGAGTAGGGTTCC-3′

*hCTNNB1* 5′-TTGAAGGTTGTACCGGAGCC-3′*;* 5′-GCAGCTGCACAAACAATGGA-3′

*hH3* 5′-GTGTCATCCATGCCAAACGG-3′; 5′-GTGGCGAGATAGCCCTCCTA-3′

### Polysome-mRNP analysis

GSCs were lysed as previously described [46, 47] with slight modifications described below. The supernatant was loaded onto a 15–50% (w/v) sucrose gradient and sedimented by centrifugation at 4 °C for 150 min at 37,000 rpm in a Beckman SW41 rotor (Fullerton, CA). Each gradient was collected into 10 fractions (1–6 = polysomes; 7–10 = mRNPs) while reading the absorbance at 254 nm, followed by the addition of 50 pg of spike-in control (luciferase control RNA, Promega L456A). Total RNA was extracted from each fraction, precipitated and the RNA quality was assessed by gel electrophoresis and spectrophotometry (ND-1000 spectrophotometer, Nanodrop Technology). The mRNAs of interest (*FMR1*, and *luciferase*) were quantified by RT-qPCR.

### Immunohistochemistry for FMRP and staining evaluation score from GBM tissues

Formalin-fixed, paraffin-embedded sections (40 µm thick) were mounted on positive charged glass slides. The series of GBM samples used for IHC (*n* = 60) differed from that used for the generation of GSCs (*n* = 28). The procedure to obtain these samples is different. While the paraffin-embedded specimens for IHC can be available just one week after surgery, bona fide GSCs require 12.8 ± 7.7 weeks to obtain sizable tumorspheres [48] and additional 4–6 months for cell grafting and in vivo studies. Only bona fide GSCs were used. For antigen retrieval to detect FMRP protein, deparaffinized and rehydrated sections were boiled in TRIS-EDTA buffer solution (pH 9) for 20 min. The slides were cooled, and endogenous peroxidase were blocked with peroxidase block buffer (citric acid 0.04 M, Na_2_HPO_4_ × 2H_2_O 0.12 M, NaN_3_ 0.03 M, and H_2_O_2_ at 1.5% v/v) for 15 min at room temperature. Then, the sections were incubated for 2 h with rabbit polyclonal antibodies against FMRP (1:200, PZ1).

The primary antibodies were visualized using the avidin-biotin-peroxidase complex method (UltraTek HRP Anti-polyvalent, ScyTek, Logan, UT) according to the instruction manual. 3,3′ diaminobenzidine was used as substrate to observe the specific antibody localization, and Mayer hematoxylin was used as a nuclear counterstain.

Staining intensity of tissue slides was evaluated independently by 2 observers (L.M.L. and M.M.), who were blinded toward the patients’ characteristics and survival. Cases with disagreement were discussed using a multi-headed microscope until agreement was achieved. To assess differences in staining intensity, an immunoreactivity scoring system was applied. FMRP expression in each specimen was scored according to the percentage of stained cells and intensity of nuclear staining. Immunohistochemistry (IHC) was scaled as 0 for no IHC signal at all, 1 for 1–30%, 2 for 31–70%, and 3 for 71–100% of tumor cells stained. The score for IHC intensity was also scaled as 0 for no IHC signal, 1 for weak, 2 for moderate, and 3 for strong IHC signals. The final score used in the analysis was calculated by multiplying the extent score and intensity score, with a maximum score equal to 9. Samples with score 0–3 were arbitrary identified as low FMRP expression, while samples with score 4–9 were identified as high FMRP expression. The specificity of FMRP detection was tested on GBM sections stained in the absence of the primary antibody and on human breast cancer sections expressing high or no FMRP levels as in [39]. At least two sections were stained for each sample, FMRP expression was highly reproducible.

### RNA extraction from GBM tissues

RNA was extracted from three, independent, 10-µm section from paraffin-embedded tissues of each patient using RNeasy FFPE Kit (QIAGEN, Milan, Italy), following the manufacturer’s protocol. Real-time PCR was performed using the KAPA SYBR FAST One-Step qRT-PCR Kit (KAPA-Biosystems, Boston, Massachusetts, USA), following the manufacturer’s protocol using the CFX96™ Real-Time PCR Detection System (Bio-Rad). Each analysis was performed in duplicate and the expression level of *FMR1* mRNA was normalized by *ACTB* mRNA.

### Lentiviral production and stable cell lines

The lentiviral expression vector to silence *FMR1* (sh*FMR1*) or non-target control (shNTC) were purchased from Sigma (Merck, pLKO.1-puro-CMV-tGFP shRNA control, and pLKO.1-puro-CMV-tGFP sh*FMR1* clone ID: TRCN0000059761 and sh*FMR1* clone ID: TRCN0000298271). The overexpression of *FMR1* mRNA was obtained using the lentiviral vector (207.pRRLsyn.PPTs.hCMV.GFP.Wpre) kindly provided by Prof. Suzanne Zukin (Albert Einstein College of Medicine, USA). These vectors also contain the GFP as reporter gene that allowed the selection of transduced cells by Fluorescent Activated Cell Sorting (FACS).

Lentiviral particles were produced by the calcium-phosphate transfection protocol in the packaging human embryonic kidney cell line 293T. Briefly, the lentiviral construct was cotransfected with pMDL, pRSV-REV and pVSV-G. The calcium-phosphate DNA precipitate was removed after 8 h by replacing the medium. Viral supernatants were collected 48 h post transfection, filtered through a 0.45 μm pore size filter, and added to GSCs in the presence of 8 μg/ml polybrene. Cells were centrifuged for 30 min at 1800 rpm. After infection, the fluorescence of transduced cells was evaluated by FACSCanto (Becton Dickinson).

### In vivo xenografts

Immunosuppressed SCID mice were anesthetized with intraperitoneal injection of diazepam (2 mg/100 g) followed by intramuscular injection of ketamine (4 mg/100 g). Animal skulls were immobilized in a stereotactic head frame and a burr hole was made 2 mm right of the midline and 1 mm anterior to the bregma. The tip of a 10 µl-Hamilton microsyringe was placed at a depth of 3 mm from the dura and 2 × 10^4^ of either sh*FMR1* GSC#148 or shNTC GSC#148 or sh*FMR1* GSC#163 or shNTC GSC#163 were slowly injected. After grafting, the animals were kept under pathogen-free conditions in positive-pressure cabinets (Tecniplast Gazzada, Varese, Italy) and observed daily for neurological signs. For survival curves, the mice were sacrificed when the body weight dropped to 80% of initial weight or at appearance of neurological signs. The mice were deeply anesthetized and transcardially perfused with 0.1 M PBS (pH 7.4) then treated with 4% paraformaldehyde in 0.1 M PBS. The head was fixed in the stereotactic head frame, the skull was removed, and the brain was blocked 2 mm posteriorly to the grafting site. The anterior block of the brain was stored in 30% sucrose buffer overnight at 4 °C and serially cryotomed at 20 µm on the coronal plane. Sections were collected in distilled water and mounted on slides with Vectashield mounting medium (Bio-Optica, Milan, Italy). Images were acquired with a laser scanning confocal microscope (LSM 500 META, Zeiss, Milan, Italy). The tumor volume was determined according to the equation: *V* = (*a*^2^ × *b*)/2, where *a* is the mean transverse diameter calculated through the tumor epicenter and *b* is the cranio-caudal extension of the tumor. The brain posterior to the grafting site was post-fixed in 10% formalin for 48 h, embedded in paraffin, and cut at 4 µm thick sections.

### Fluorescence Microscopy and Immunohistochemistry on brain xenografts

For immunofluorescence, coronal sections of the brain were blocked in PB with 10% BSA, 0.3% Triton X-100 for 45 min. Sections were incubated overnight at 4 °C with primary antibodies in PB with 0.3% Triton X-100 and 0.1% normal donkey serum (NDS). Polyclonal antibodies used was Ki67 (1:500, Thermo Fisher Scientific, Cod. RM9106S1). For detecting brain microvessels, sections were incubated overnight at 4 °C in PB with 0.3% Triton X-100 and 0.1% NDS with Lectin from Lycopersicon esculentum (tomato) biotin conjugate (1:500, Sigma-Aldrich, St. Louis, MO) together with primary antibodies. Slices were rinsed and incubated in PB containing 0.3% Triton X-100 with secondary antibodies for 2 hours at RT. For lectin staining, sections were incubated for 2 h at RT in PB containing 0.3% Triton X-100 with streptavidin protein, DyLight 405 conjugate or streptavidin Alexa Fluor^®^ 647 conjugate (1:200, Thermo Fisher Scientific, Waltham, MA, USA). Before mounting, slices were incubated with DAPI (1:4000; Sigma-Aldrich) for 10 min. Immunofluorescence was observed with a laser confocal microscope (SP5; Leica) and images were acquired. Image analysis was performed with Leica Application Suite X software. The MIB-1 staining index was determined as the percentage of Ki67-positive cells relative to the total number of cells in high power fields (400×). In each tumor specimen, at least 1500 tumor cells were counted. The number of neoformed vascular structures was assessed in lection stained coronal sections of xenografted brains through the tumor epicenter.

### Proliferation and cell viability assays

Cell viability: shNTC or sh*FMR1* transduced GSCs were plated at density of 2 × 10^3^/ml in 96 well plates in triplicate. Cell viability was monitored by counting the cells and confirmed by using the CellTiter-Blue™ Viability Assay (Promega). Cell proliferation: was evaluated by Bromo-2′-deoxyuridine (BrdU) incorporation using BrdU Cell proliferation ELISA kit (Abcam, Cod. ab126556) following the manufacturer's instructions. The motility of transduced GSCs was evaluated by plating in Corning FluoroBlok^TM^ Multiwell Inserts System (Corning Life Sciences, Tewksbury, MA), according to the manufacturer's instruction. Briefly, 3x10^3^ cells were added to the upper chambers in stem cell medium without growth factors (GFs). GF completed medium was used as chemoattractant in the lower chambers. The plates were incubated for 48 h at 37 °C, after which the fluorescent dye calcein acetoxymethylester (calcein AM, Life Technologies) was added to the lower chamber for 30 min. The cell viability indicator calcein AM is a non-fluorescent, cell-permeant compound that is hydrolyzed by intracellular esterases into the fluorescent anion calcein and can be used to fluorescently label viable cells before microscope observation. The number of migrated cells was evaluated by counting the cells after imaging acquisition using a fluorescence microscope (Nikon Eclipse TS100).

### RNA-seq and statistical analysis

To study the FMRP-regulated pathways in GBM, we compared existing datasets derived from human glioblastoma and mouse brain. For the human dataset we interrogated the genes mutated in GBM listed in the cBioPortal for cancer genomics (http://cbioportal.org) selecting six studies of glioblastoma stemming from The Cancer Genome Atlas (TCGA, four studies), Mayo Clinic (one study in 2019, Columbia University (one study in 2019) for a total of 1184 patients. Genes that were mutated in at least 1% of all patients were considered for further analysis (1077 genes are mutated). For the mouse datasets, FMRP target mRNAs in mouse hippocampus derived from two CLIP-seq studies were selected. For each of the two relevant studies [49, 50], the log2 of the count of sequence tags (indicating the FMRP binding strength) was z-score normalised. Genes that had, on average, a z-score of at least +1 (top ~37% of FMRP crosslinked mRNA targets) were considered (550 genes), and their human homologs were identified using the vertebrate homology table of the Mouse Genome Informatics (MGI) database (http://www.informatics.jax.org/downloads/reports/index.html#homology). The overlap (52 genes) of both gene lists (1077 and 550) was screened for enrichment in the PANTHER pathway database using the tool provided (pantherdb.org); the reference list was set to all genes that were reported either in cBioPortal/GBM or in both of the CLIP studies. The overlap (73 genes) of the genes frequently mutated in GBM (see above) and the list of genes associated with autism (1023 genes, Simons Foundation Autism Research Initiative, SFARI, https://gene.sfari.org) was analyzed in the same way.

RNA-seq was performed on control and *FMR1*-silenced cells. GSCs stably transduced with shNTC or sh*FMR1* vectors were expanded and GFP-positive cells selected by FACS. Total RNA was extracted from control or *FMR1* silenced cells using TRIzol reagent (Life Technologies). Three independent cell expansion and FACS selection rounds were performed. The quality and quantity of the RNA were measured by spectrophotometry (Nanodrop, Thermo Fisher Scientific), and RNA integrity was verified on a Bioanalyser (Agilent). If quantity (minim 100 ng) and quality (RIN factor of 5) of the RNA was deemed sufficient mRNA expression profile (RNA-seq) was analyzed by Illumina HiSeq at the Genomic Technologies Facility (GTF) of the University of Lausanne, Switzerland (www.unil.ch/gtf). Reads were aligned to the human genome (GRCh38.86) and assigned to the respective mRNAs using the Cufflinks algorithm; differential expression between samples has been analyzed by the DESeq module of the Bioconductor suite. To gauge the effect of *FMR1* silencing, the log2(FPKM) of all samples for a given cell line was compared by principle component analysis (PCA, function prcomp() in R), plotting the first and second dimension (package ggplot). Human genes linked to WNT-related pathways (excluding “cell polarity”) were downloaded from geneontology.org (441 genes, 370 of which were detected in the RNA-seq experiment in the three cell lines). Gene Set Enrichment Analysis (GSEA) was performed as follows: the Wnt-associated genesets (N=70) was downloaded from the Molecular Signature Database (https://www.gsea-msigdb.org/gsea/msigdb/). The log fold change (logFC) of gene expression was calculated comparing sh*FMR1* and the shNTC GSCs transcriptome for a total of 16572 genes, which were then used to run pre-ranked GSEA (GSEA, https://www.gsea-msigdb.org/gsea/index.jsp) using weighted enrichment statistics and 1000 random sample sets permutation.

### RNA stability assay

T98G cells were silenced for FMRP using *FMR1*-specific siRNAs from Life Technologies (Carlsbad, CA, USA) (AM 16708, ID 10824, 10919, and 11010). As a nonspecific control, a scrambled siRNA was used (Cod. 4390843; Life Technologies). siRNA was transfected into T98G cells using Lipofectamine RNAiMAX (Life Technologies, Cod. 13778100), according to the manufacturer’s instructions. Transfections were carried out with 90 pmol of siRNA, and cells were used for the experiment after 48h. T98G control cells and T98G *FMR1-silenced* cells were treated at *t* = 0 with Actinomycin D (1 mg/ml) for 0, 2, 4, 6, 8 h. RNA was extracted and RT‐qPCR performed as previously described.

### Quantification and statistical analysis

Data quantification has been described in the figure legends and in some of the methods in this section. Statistical analysis was performed using GraphPad-Prism 5 software (Graph Pad Software, San Diego, CA) and MedCalc version 10.2.0.0 (MedCalc Software, Mariakerke, Belgium). Comparisons between the two conditions, shNTC and sh*FMR1*, were performed using two-sample two-tailed Student’s *t*-tests or One-sample *t*-test. Growth curves were analyzed using two-way ANOVA. Correlation was assessed by Pearson regression analysis. Comparison of categorical variables was performed by chi-square statistic, using the Fisher’s exact test. Kaplan-Meier survival curves were plotted and differences in survival between groups of patients were compared using the log-rank test. Statistical comparison of continuous variables was performed using Mann-Whitney U-test. Significance was denoted as *P < 0.05, **P < 0.01, ***P < 0.001. Error bars represent the standard error of the mean (SEM).

### Reporting summary

Further information on research design is available in the Nature Research Reporting Summary linked to this article.

## Results

### FMRP is upregulated in GSCs due to enhanced *FMR1* mRNA translation

**Fig. 1:**
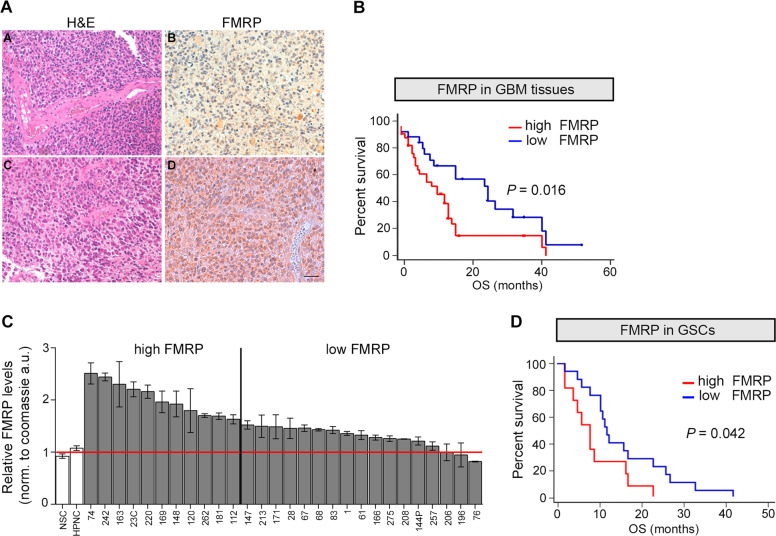
FMRP is upregulated in GBM and is associated with worst outcome. **A** Representative histological section from GBM patients stained with hematoxylin/eosin (H&E) (**A**, **C**) and after immunostaining for FMRP (**B**, **D**). FMRP is mostly observed as diffuse cytoplasmic staining; most cells in **D**, but only few in **B**, show FMRP staining. Scale bar, 50 µm. **B** Kaplan–Meier survival curve of GBM patients stratified by FMRP expression. Low FMRP expression (blue line; 25 cases; median survival 26 months) was significantly associated to a better patient overall survival (OS) (*P* = 0.016; HR 0.45; 95% CI from 0.24 to 0.86) respect to high FMRP levels (red line; 35 cases; median survival 12 months). **C** Quantification of FMRP expression in 28 GSCs. Values were normalised for Coomassie staining and are shown relative to FMRP levels in NSC and HPNC10 control cells, the average of which is set to 1 (mean ± SEM, technical replicates for each GSC line). **D** Kaplan–Meier survival curve of GBM patients showed that low FMRP expression (blue line; 17 GSC lines; median survival 12 months) was significantly associated to a better OS (*P* = 0.042; HR 0.389; 95% CI from 0.15 to 0.98) respect to high FMRP levels (red line; 11 GSC lines; median survival 8 months).

To address whether FMRP is involved in the biology of GBM, we investigated its expression in different GBM biopsies. FMRP levels were analyzed by immunohistochemistry (IHC) in GBM biopsies derived from 60 patients (Fig. 1A), using a specific antibody (Fig. S1A and see also Materials and Methods). FMRP expression varies between GBM samples and high FMRP expression correlates with poor survival rates (see the Kaplan–Meier analysis in Fig. 1B). The poor prognosis of GBM is attributable to treatment failure and eventual disease relapse, a process in which GBM stem-like cells (GSCs) play a crucial role [2]. The investigation of GSCs biology is therefore an imperative need to find an effective therapy for GBM, yet very little is known about the actions of specific RNA-binding proteins in the dysregulation of the GSC transcriptome.

GSCs can be isolated and cultured in vitro [9, 51, 52], and secondary tumors can be generated in vivo through orthotopic transplant xenografting into mice [53, 54]. To assess the possible role of FMRP in GBM, we first analyzed FMRP expression in 28 patient-derived GSCs (not generated from the 60 GBM described above, see Materials and methods) in vitro (Supplementary Table 1). Western blot analysis revealed increased FMRP expression in GSCs compared to healthy neural stem cells isolated from human adult olfactory bulb (NSCs) and fetal brain (HPNC10), two cell types with a basal expression of FMRP [55] (Fig. 1C and Fig. S1B). Importantly, high FMRP levels in individual GBM-derived GSCs correlate with patients’ overall poor survival (Fig. 1 D and Supplementary Table 1), similarly to the observations in the GBM biopsies (Fig. 1B).

Surprisingly, we found that *FMR1* mRNA levels were lower in GSCs compared to control cell lines (Fig. S2A), with decreased levels of *FMR1* mRNA associated with poor survival (Fig. S2B). Furthermore, the analysis of *FMR1* mRNA levels in GBM tissues used for the IHC experiment, revealed a positive association between *FMR1* mRNA expression and patient survival rates (Fig. S2C). Consistently, a meta-analysis of *FMR1* mRNA expression in the TCGA GBM database revealed that patients with high mRNA expression had a better prognosis compared to those with low expression (Fig. S2D).

The discrepancy of the correlation between patients’ survival and level of *FMR1* mRNA or FMRP prompted us to evaluate *FMR1* mRNA translational efficiency in seven different GSC lines that expressed high (#112, #148, #163, #169), medium-high (#83, #144P, #206) FMRP levels and, very low (#83, #112, #144P, #163) or low (#148, #169, #206) *FMR1* mRNA levels. This was achieved by performing an analysis of the relative distribution of *FMR1* mRNA on a polysome-mRNP sucrose gradient (Fig. S3E) specifically in those fractions corresponding to actively translating polysomes and silent mRNPs (percentage of mRNA on polysomes, PMP), as previously described [39, 47]. We found that FMRP protein levels were positively correlated with the PMP and negatively correlated with *FMR1* mRNA levels (Fig. S2F). These findings might explain that in those cells where the level of *FMR1* mRNA is low, the efficiency of translation is high, ultimately producing high levels of FMRP contributing to tumor aggressiveness in GBM.

### FMRP downregulation reduces tumor growth and tumor vascularity in vivo

Orthotopic xenografts of GSCs provide an invaluable in vivo model to investigate the factors involved in malignant growth and brain invasion [56]. To analyse if modulation of *FMR1* expression could affect tumor growth in vivo we used xenografts of GSCs in which we reduced FMRP expression using lentiviral vectors expressing either shRNA against the *FMR1* mRNA (sh*FMR1*) or a non-target control (shNTC) in three GSC lines that are characterized by high FMRP expression and poor prognosis (#148, #163 and #169; Fig. 1C and Supplementary Table 1). sh*FMR1* transduction reduces FMRP and *FMR1* mRNA levels by more than 50% (Fig. S3A, B). To exclude an off-target effect, we generated a second knockdown GSC (#163) using a different sh*FMR1* molecule. Consistent with previous observations, also in this case, a specific and efficient FMRP downregulation was observed (Fig. S3C).

**Fig. 2:**
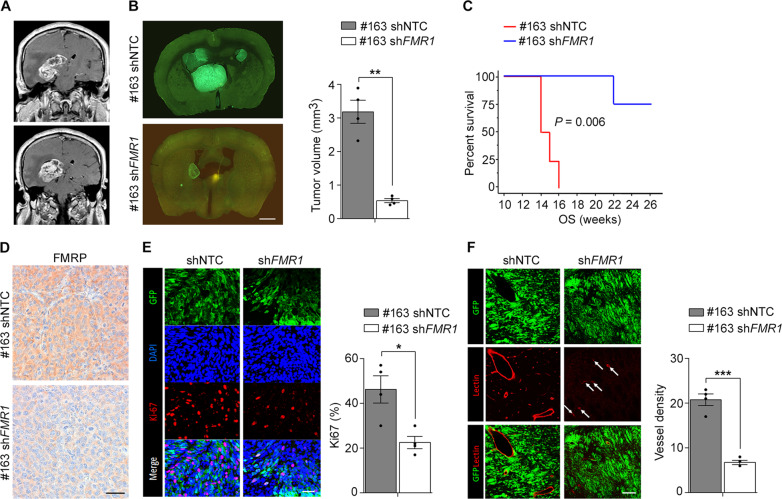
FMRP levels affect tumor cell proliferation and vascularity in brain xenografts. **A** Gadolinium-enhanced T1-weighted coronal magnetic resonance of the patient the GSC#163 line was generated from. **B** The left panel represents coronal brain sections of shNTC GSC#163 and sh*FMR1* GSC#163 grafts, where the latter showed remarkable tumor growth reduction. Right panel shows tumor volume quantification (mean ± SEM, *n* = 4/groups, **P < 0.01, Student’s *t*-test). Scale bar, 800 µm. **C** Kaplan-Meier plot showing the survival of mice grafted with shNTC GSC#163 (red line; *n* = 4 mice; median survival 15 weeks) and sh*FMR1* GSC#163 (blue line; *n* = 4 mice; median survival 24 weeks) (*P* = 0.006, log-rank test). **D** Immunohistochemistry with anti-FMRP antibody shows a lower signal in GSC #163 sh*FMR1* xenografts compared to GSC #163 shNTC xenografts. Scale bar, 40 µm. **E** Left panel, representative anti-Ki67 immunohistochemistry (red) and DAPI (blue) of tumors derived from shNTC GSC#163 and sh*FMR1* GSC#163 grafts, expressing GFP (green). Scale bar, 30 µm. Right panel, quantification of Ki67 labeling index. (mean ± SEM, *n* = 4/groups, *P < 0.05, Student’s *t*-test). **F** Left panel, brain microvessels (red) were detected with Lectin. Scale bar, 45 µm. Right panel, quantification of the number of microvascular structures per microscopic field (mean ± SEM, *n* = 4/groups, ***P < 0.001, Student’s *t*-test).

The GSC #163 cells were derived from a patient who had a GBM with the tendency to grow around the lateral ventricles, as shown by the brain magnetic resonance imaging (MRI) (Fig. 2A). We implanted green fluorescent protein (GFP)-expressing shNTC GSC #163 or sh*FMR1* GSC #163 onto the right striatum of NOD-SCID immunodeficient mice. Fourteen to sixteen weeks after grafting, mice injected with shNTC GSC #163 harbored large tumors that invaded extensively the fimbria and grew into the lateral ventricles and third ventricle, which resembles the MRI from the patient. Of note, mice carrying sh*FMR1* GSC #163 transplants had much smaller brain tumors (Fig. 2B). Moreover, mice bearing sh*FMR1* GSC #163 grafts had better survival rates compared to shNTC GSC #163 (Fig. 2C). Importantly, these results were corroborated by brain xenografts using a second GSC line, namely GSC #148 (Fig. S4A, B). Immunostaining for FMRP on brain sections from the orthotopically injected mice confirmed a reduced expression of FMRP (Fig. 2D).

Given the decreased tumor growth seen in the *shFMR1* GSC xenografted mice, the impact of FMRP on GSCs proliferation and tumor cell proliferation was analyzed in vivo using the Ki67 labeling index. Cell proliferation was significantly decreased in sh*FMR1* GSC#163 tumors compared to shNTC grafts (Fig. 2E). Moreover, lycopersicon esculentum lectin staining of the vascular network indicated that microvessel density, important to maintain cell proliferation, was significantly reduced in sh*FMR1* GSC #163 compared to shNTC GSC #163 xenografts (Fig. 2F). Taken together, these findings indicated that downregulation of FMRP expression reduced tumor growth and proliferation in vivo.

### FMRP is required for GSC viability and proliferation

**Fig. 3:**
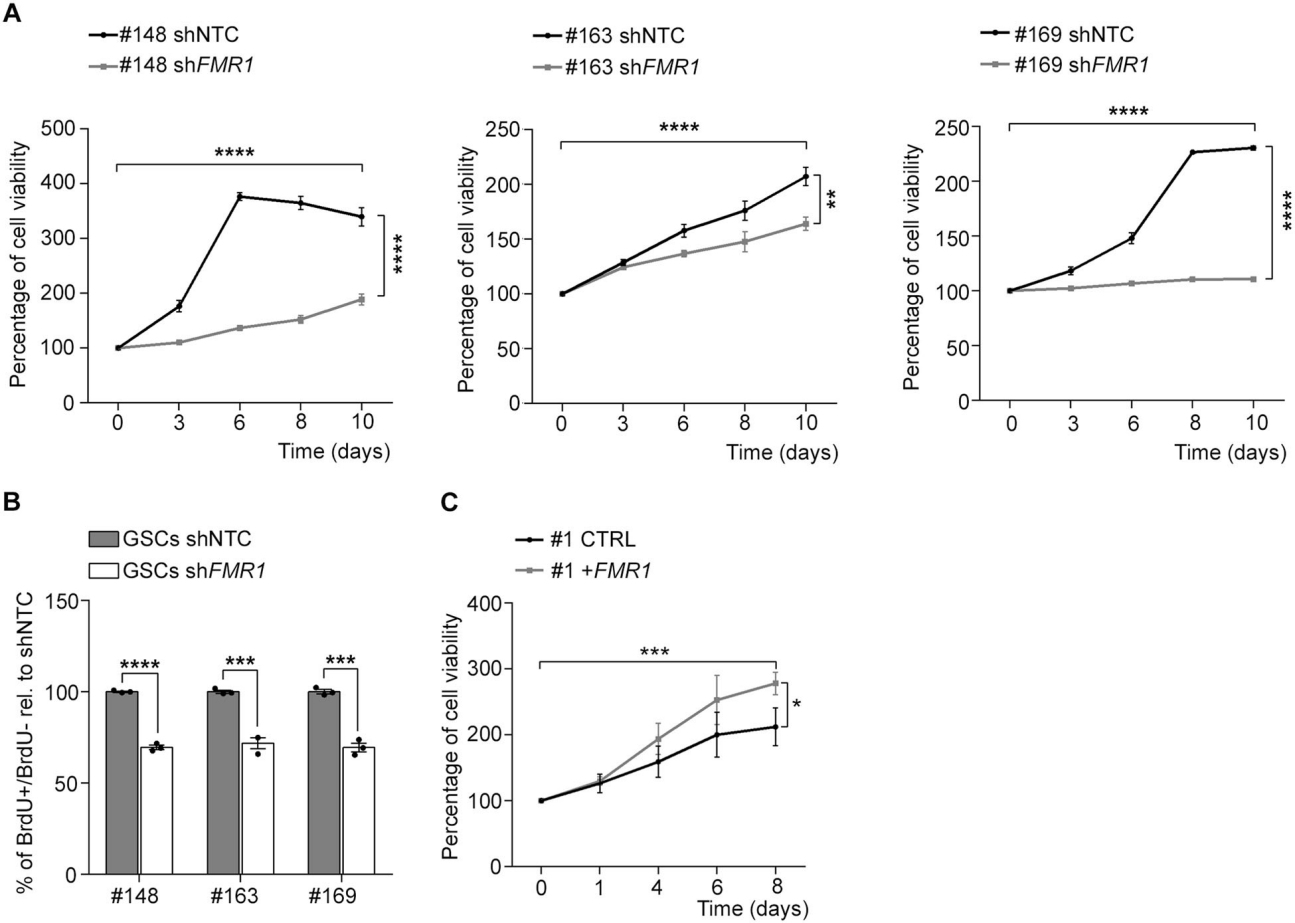
Reduction of FMRP impairs proliferation of GSCs. **A** Growth curves of GSCs transduced with either shNTC or sh*FMR1* (as indicated on top of each graph). The percentage of cell viability is plotted against days in culture (mean ± SEM, *n* = 3, two-way ANOVA). **B** The percentage of BrdU incorporation in the sh*FMR1* compared to shNTC GSCs (mean ± SEM, *n* = 3, ***P < 0.001, Student’s *t*-test). **C** Growth curves of GSC#1 transduced with the empty vector (CTRL) or with the vector overexpressing FMRP (+*FMR*1). The percentage of cell viability is plotted against days in culture (mean ± SEM, *n* = 3, *P < 0.05, ***P < 0.001, two-way ANOVA).

To further validate a role for FMRP in conferring an aggressive proliferation potential to GSCs, we evaluated the proliferation ability in the three *FMR1*-silenced cell lines (#148, #163, and #169) in vitro (Fig. 3). When FMRP expression was reduced by 60–80% (Fig. S3), cell viability was significantly decreased (Fig. 3A). Consistently, using the BrdU proliferation assay, we found a reduction by 30–40% in the number of BrdU-positive cells in sh*FMR1* GSCs compared to the shNTC GSCs (Fig. 3B). Furthermore, to exclude an off-target effect, we performed an independent experiment using a second sh*FMR1* molecule, confirming a reduction of cell growth in vitro (Fig. S5A). Finally, to further corroborate the role of FMRP in cell proliferation, we overexpressed FMRP in a GSC line that is characterized by low FMRP expression and better prognosis (#1; Fig. 1C, Fig. S5B, C and Supplementary Table 1). Under these conditions, FMRP upregulation induced a significant increase in the GSC #1 proliferation ability (Fig. 3C).

### FMRP regulates WNT canonical and non-canonical pathways in GSCs

The presence of GSCs in GBM is the major cause of the high intertumoral heterogeneity [5], therefore the identification of dysregulated molecular mechanism/s in GSCs might be key to understand a molecular signature in GBM and develop an effective therapy.

FMRP regulates a large group of neuronal mRNAs also involved in cancer progression [37]. Furthermore, FXS is the most common form of autism (ASD) and recent genome-wide exome sequencing studies of de novo variants in genes implicated in intellectual disabilities and cancer have begun to uncover considerable overlap in the number of risk genes [57, 58].

**Fig. 4:**
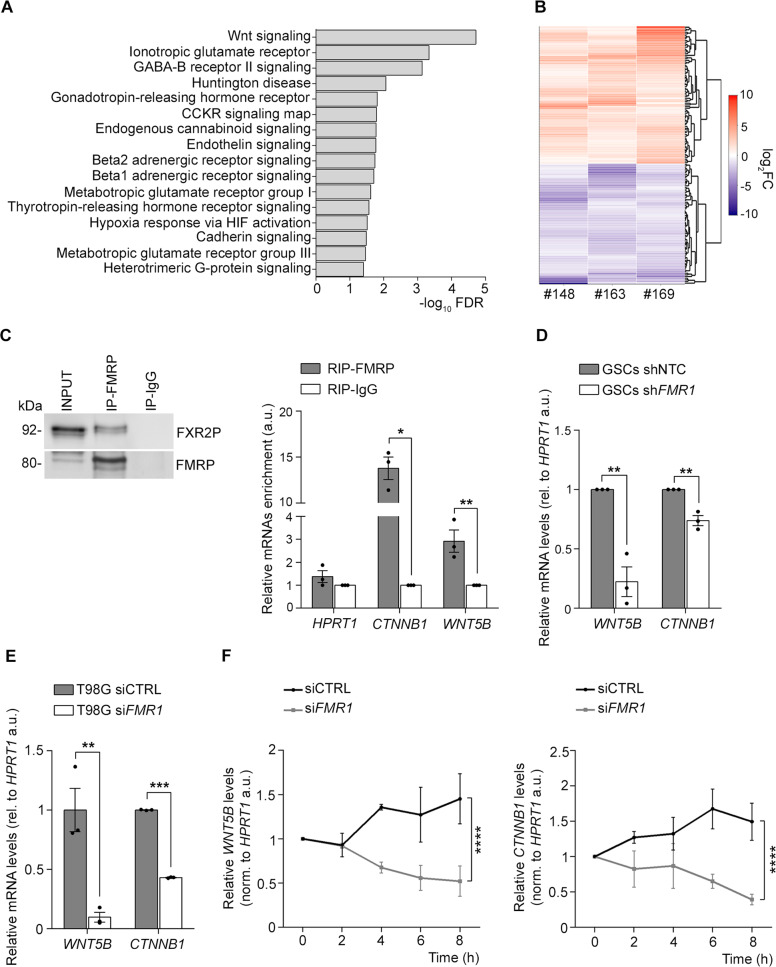
FMRP modulates the WNT pathways regulating the *WNT5B* and *CTNNB1* mRNA metabolism. **A** Enrichment analysis of known FMRP target mRNAs and mutated in GBM according to The Cancer Genome Atlas. Shown are pathways that are significantly enriched in the PANTHER database (FDR < 0.05). **B** Cluster heat map represents the log_2_ fold change (FC) for the 156 genes differentially expressed in all the three sh*FMR1* GSCs. Values on a log_2_ scale are color-coded as indicated on the right. **C** Left panel, representative western blot of total extract (input), FMRP immunoprecipitation (IP-FMRP) and mock immunoprecipitation (IP-IgG). FMRP and its interactor FXR2P are detected. Right panel, *HPRT1*, *CTNNB1*, and *WNT5B* mRNAs were quantified by RT-qPCR. Shown is the enrichment immunoprecipitation/total, relative to *H3* mRNA (mean ± SEM, *n* = 3, *P < 0.05, **P < 0.01, Student’s *t*-test). *HPRT1* mRNA was used as a negative control; *CTNNB1* mRNA is also a well-known FMRP target and was used as a positive control. **D** The histogram represents *WNT5B* and *CTNNB1* mRNA levels quantified in shNTC and sh*FMR1* #148, #163, and #169 GSC cell lines. *HPRT1* mRNA was used as normaliser (mean ± SEM, *n* = 3, **P < 0.01, Student’s *t*-test). **E** *WNT5B* and *CTNNB1* mRNA levels in CTRL siRNA (siCTRL) and *FMR1* siRNA (si*FMR1*) T89G cells detected by RT-qPCR (mean ± SEM, *n* = 3, **P < 0.01, ***P < 0.001, Student’s *t*-test). **F** mRNA stability assay in CTRL siRNA (siCTRL) and *FMR1* siRNA (si*FMR1*) T89G cells. RNA was isolated at the indicated time points after Actinomycin D treatment and the stability of *WNT5B* and *CTNNB1* mRNAs was analysed by RT-qPCR (mean ± SEM, *n* = 3, ***P < 0.001, ****P < 0.0001, two-way ANOVA).

The analysis of the overlap between the genes associated with autism (1023 genes, Simons Foundation Autism Research Initiative, SFARI, https://gene.sfari.org) and the genes mutated in GBM (cBioPortal database, http://www.cbioportal.org, the top 1077 genes were considered, see Materials and methods) highlighted the WNT signalling as one of the most enriched pathways (Fig. S6A). Moreover, we showed that the WNT signalling is also the most significantly enriched in the overlap between the FMRP target genes identified by CLIP-seq ([49, 50]; the top 550 genes were considered, see Materials and methods) and the genes frequently mutated in GBM. These results suggest that the WNT pathway may mediate the effect of FMRP on GBM aggressiveness (Fig. 4A). In addition, the list contains several synaptic pathways such as glutamatergic and GABAergic signalling, suggesting that FMRP might regulate the connection of GBMs into neuronal networks, which is beneficial for tumor growth [59]. Since the WNT pathway might underlie the proliferation phenotype observed in vitro and in vivo (Figs. 2 and 3), we aimed at understanding if and how FMRP affects this mechanism in GSCs. To investigate which protein/s involved in WNT signalling alter expression upon FMRP depletion, we performed a transcriptome profiling of the three GSCs (#148, #163, and #169), comparing sh*FMR1* versus shNTC GSCs (Fig. S6B). Transcripts corresponding to a total of 16572 genes were identified. *FMR1* silencing in all three cell lines had drastic effects at the overall transcriptome level (Supplementary Table 2). Notably, 156 genes consistently showed the same differential expression in all three sh*FMR1* GSCs compared to shNTC cells (Supplementary Table 3). Among these differentially expressed genes, 52% (81/156) were upregulated and 48% (75/156) were downregulated in sh*FMR1* GSCs (Fig. 4B). We found a downregulation of mRNAs expression involved in WNT signalling, including two key molecules of this pathway, *WNT5B* and its main effector *β-Catenin* (*CTNNB1*) (Supplementary Table 4). Gene Set Enrichment Analysis (GSEA) using a collection of genesets representative of the WNT signalling (*N* = 70; see Materials and methods for details) confirmed that the significant (*p* < 0.05; Supplementary Table 5) enriched WNT-related genesets were prevalently associated with negative ‘normalized enrichment scores’ (NES) in sh*FMR1* GSCs (Fig. S6C), thus confirming the inhibition of WNT pathway by *FMR1* silencing in all the three GSC lines. Immunoprecipitation of FMRP demonstrated that *WNT5B* and *CTNNB1* mRNAs are targets of FMRP (Fig. 4C), and evaluation of the corresponding mRNA level in the 3 GSC lines revealed a significant downregulation in their expression upon FMRP reduction (Fig. 4D), further validating the RNA-seq data. To further investigate the effect of FMRP on these mRNAs in glioblastoma, we detected *WNT5B* and *CTNNB1* mRNA levels in an aggressive commercial glioblastoma cell line, namely T98G. We showed a significant decrease of these two mRNAs upon FMRP reduction, further supporting and validating the RNA-seq dataset (Fig. 4E).

FMRP has been shown to regulate mRNA metabolism also at the level of mRNA stability [24, 26]. As *WNT5B* and *CTNNB1* mRNAs are part of the FMRP complex and their expression decreases upon FMRP reduction, we evaluated the stability of these two mRNAs in cells expressing different levels of FMRP. A faster decay of *WNT5B* and *CTNNB1* mRNAs was detected when FMRP was decreased (Fig. 4F) showing an involment of FMRP in regulating the stability of these mRNAs.

**Fig. 5:**
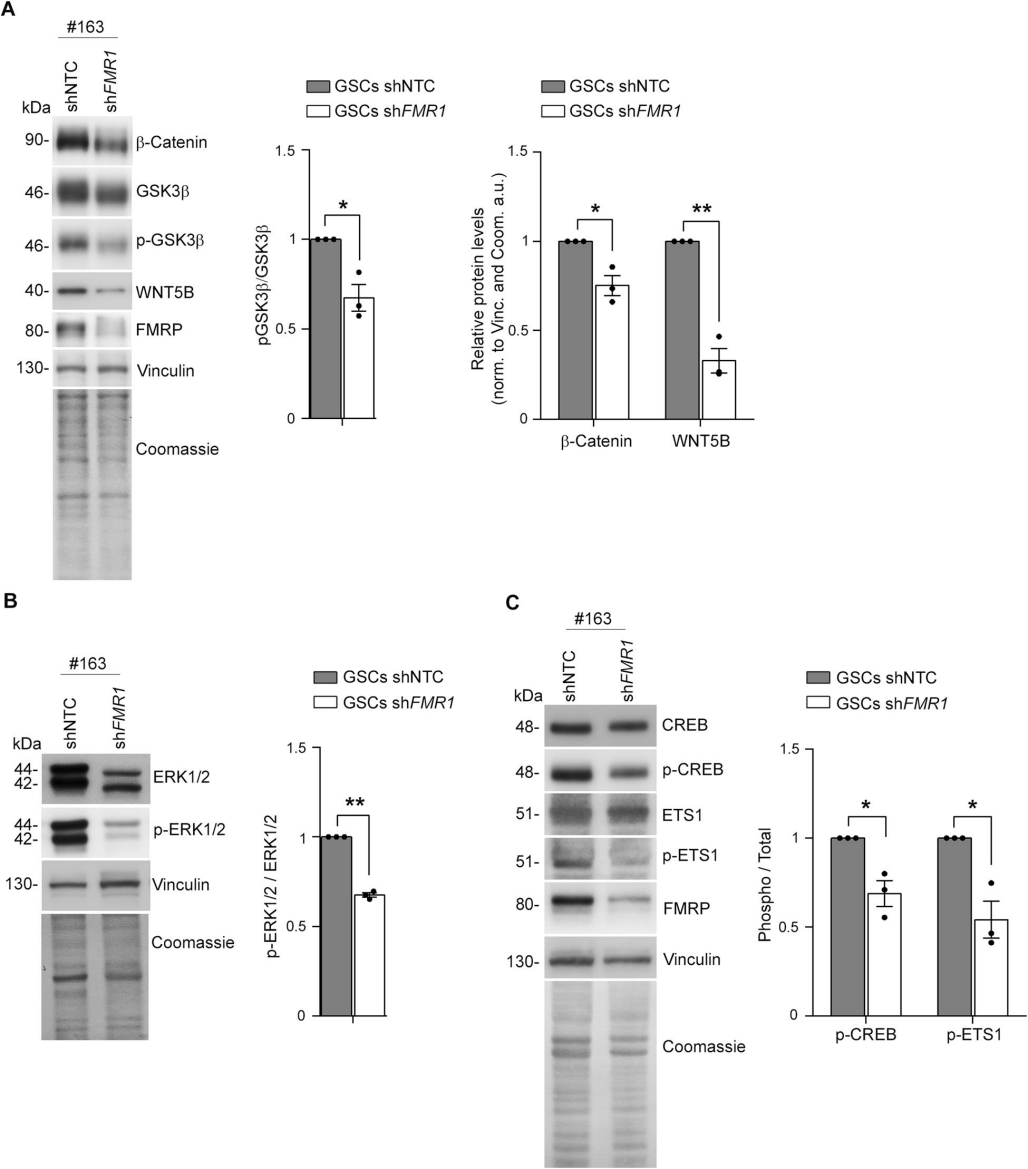
FMRP modulates the WNT canonical and non-canonical pathways. **A** Left panel shows representative western blot of β-Catenin, GSK3β, and p-GSK3β levels in GSCs transduced with shNTC and sh*FMR1*. Vinculin and Coomassie staining were used as normalisers. Right histogram represents the quantification of the biological replicates of p-GSK3β, β-Catenin and WNT5B (#148, #163, and #169 GSCs). p-GSK3β was normalised over total GSK3β levels (mean ± SEM, *n* = 3 independent cell lines, each data point averaged from three FACS sortings, **P < 0.01, ***P < 0.001, One-sample *t*-test). **B** Left panel shows representative western blot of ERK1/2 and p-ERK1/2 levels in GSCs transduced with shNTC and sh*FMR1*. Vinculin and Coomassie staining were used as normalisers. Right histogram represents the quantification of the biological replicates (#148, #163, and #169 GSCs). p-ERK1/2 was normalised over total ERK1/2 levels (mean ± SEM, *n* = 3 independent cell lines, each data point averaged from three FACS sortings, ***P < 0.001, One-sample *t*-test). **C** Left panel shows representative Western blot of CREB, p-CREB, ETS1 and p-ETS1 levels in GSCs transduced with shNTC and sh*FMR1*. Vinculin and Coomassie staining were used as normalisers. Right histogram represents the quantification of the biological replicates (#148, #163 and #169 GSCs). Phosphorylated proteins were normalised over total protein levels (mean ± SEM, *n* = 3 different cell lines, each data point is the average of three independent FACS sortings, *P < 0.05, **P < 0.01, One-sample *t*-test).

**Fig. 6:**
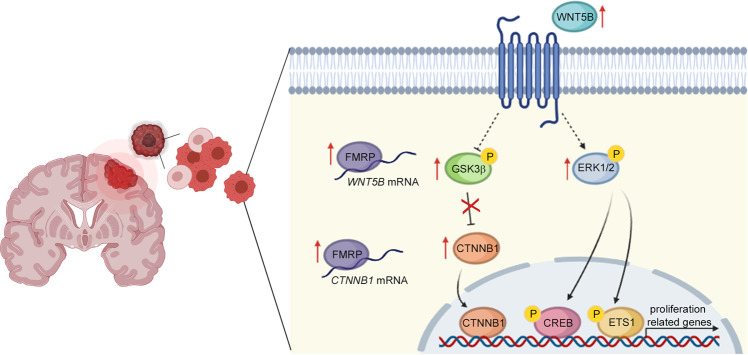
Molecular model of FMRP-mediated regulation in GSCs. FMRP is upregulated in GSCs and regulates the expression of *WNT5B* and *CTNNB1* mRNAs, pivotal in stem cells maintenance. Increased levels of FMRP lead to the aberrant activation of the WNT canonical and non-canonical signalling pathways affecting the transcription of genes related to cell proliferation.

WNT5B is upregulated in several cancer types, knowning to activate both canonical WNT/β-Catenin and non-canonical WNT signalling [60]. Through the canonical pathway, WNT inhibits glycogen synthase kinase 3β (GSK3β) leading to an increase of intracellular β-Catenin. The non-canonical WNT signalling can act as positive regulator of ERK1/2 pathway which ultimately impacts on cell proliferation [61, 62]. Consistently with a reduction of *WNT5B* mRNA, we have also observed a reduction of WNT5B protein (Fig. 5A). We next assessed GSK3β and ERK1/2 activity in our GSCs. We found that FMRP silencing increased GSK3β activity, likely caused by the observed reduction of inhibitory phosphorylation at Ser9, with a consequent downregulation of the transcription factor β-Catenin (Fig. 5A). Moreover, we found a significant decrease in ERK1/2 phosphorylation (Fig. 5B). Among the transcription factors regulated by ERK1/2 and involved in cell proliferation, CREB and ETS1 are known to modulate malignant features of glioma cells [63] controlling transcription of GBM genes [64, 65]. Consistently, we observed a decrease of CREB and ETS1 phosphorylation in GSCs silenced for *FMR1* mRNA (Fig. 5C). Overall, these findings suggest that absence of FMRP might be protective toward GBM progression by inhibiting the proliferation of GSCs regulated by both WNT canonical and non-canonical pathways (see model, Fig. 6).

## Discussion

The present study provides evidence of a direct involvement of FMRP in the GSC biology and shows that high levels of FMRP correlate with aggressive behavior of the GSCs in vitro and in vivo, and hence with poor prognosis. One of the major challenges in GBM research is to integrate the data of comprehensive genomic signatures with molecular/cellular mechanisms to understand whether and how the identified genes work alone or in network with other molecules. In the complex scenario of gene expression regulation, RNA-binding proteins (RBPs), like FMRP, are key players. They can dramatically affect the fate of a wide spectrum of target RNAs acting at different levels of RNA metabolism, contributing to tumor initiation and growth [66]. Aberrant expression of genes encoding for RBPs has been described to correlate with tumor progression, treatment responses and prognosis in various human cancers [67–69]. A study of gene expression profile across more than 2000 patient samples spanning 9 cancer types from the TCGA database revealed a unique signature for a subset of RBPs classified as strongly upregulated RBPs (SURs), in at least two-thirds of the cancer types analyzed, including brain cancer. Importantly, FMRP was included among the 31 cancer-related SURs [70]. Further, FMRP regulates a large subset of mRNAs, which play a major role in brain and cancers. Moreover, several of the FMRP protein interactors [38] and validated mRNA targets [37] identified so far are either involved or mutated in cancers suggesting that FMRP-centered regulatory networks are disturbed.

In the context of GBM, the number of well-characterized oncogenic RBPs is still relatively small [71]. According to the cancer stem cell hypothesis, the poor clinical outcome of GBM is driven in large part by the GSCs [2, 48]. These cells, which reside in specific niches of the GBM, show powerful capabilities of invasion, are able of initiating and sustaining tumor growth, causing tumor recurrences and therapeutic resistance [12, 13]. Here we demonstrated, in GSC lines derived from three different GBM patients, that reduction of FMRP results in a considerable inhibition of GSC proliferation capability. This suggests that FMRP could be a promising pharmacological target regulating common pathological pathways in GSCs.

To reproduce tumor growth and mimic the clinical pattern of tumor behavior, we used a xenograft mouse model, demonstrating in vivo that the silencing of *FMR1* gene affects GSC growth capability. Mice transplanted with sh*FMR1* GSCs survived significantly longer compared to controls due to reduced cell proliferation and tumor angiogenesis. These findings are consistent with a role of FMRP in tumor cell aggressiveness regulation, as previously described in other cancer types [15, 16, 18, 39].

To investigate the mechanisms by which FMRP affects the biology of GSCs, we performed transcriptome profiling in three GSCs derived from different patients with GBM, revealing that WNT-mediated signalling is reduced upon *FMR1* silencing. Interestingly, previous observations have shown the WNT/β-Catenin signalling involved in cell proliferation, as a potential therapeutic target in GBM [72, 73]. However, how the dysregulation of WNT pathway(s) might be involved in modulating GSCs’ activity remains largely unknown. Here we found that FMRP stabilises the *WNT5B* and *CTNNB1* mRNAs, leading to an increase of their expression in GSCs and ultimately resulting in aberrant cell proliferation. Consequently, upon *FMR1* silencing, we showed a significant downregulation of the WNT canonical and non-canonical signalling pathways. Indeed, we showed a decrease in the WNT5B and β-Catenin expression upon FMRP reduction in GSCs. Furthermore, FMRP silencing results in increased GSK3β activity leading to a consequent reduction of β-Catenin levels, a critical regulator of the WNT canonical pathway that induces the activation of gene transcription. Of note, we also demonstrated an inhibition of the non-canonical WNT pathway by a decrease of ERK1/2 phosphorylation. Aberrant kinase signalling is a common feature of all GBM types and high levels of p-ERK1/2 are usually associated with shorter overall survival and higher proliferation of tumor cells [74]. Moreover, a therapeutic strategy with temozolomide combined with ERK1/2 inhibitors has been proposed to improve the treatment of glioma cells [75]. Interestingly, our data indicate that among the mRNAs downregulated upon *FMR1* silencing in GSCs, around 70% are targets of the transcription factors β-Catenin, ETS1, and CREB (data not shown), which act downstream of WNT signalling. These results highlight a role of FMRP in GBM aggressiveness through the regulation of the WNT pathway, inducing the expression/activation of different transcription factors that modulate a subset of genes involved in cell proliferation.

Notably, it has been shown that activity of GSK3β and ERK1/2 are dysregulated in different neurodevelopmental disorders, including FXS [76–78]. These findings suggest that the same pathways might provide susceptibility or protection to different diseases in the same individual.

In agreement with our results, FMRP was reported to be part of a feedback loop between a circular RNA and the transcription factor HOXC8 ultimately facilitating the proliferation of GSCs via p53 [79]. Our findings highlight that there are different ways through which FMRP regulates proliferation of GSCs, via different and possibly independent mechanisms. FMRP thus emerges as master regulator of a large group of mRNAs regulated via the WNT signalling pathway.

The absence of FMRP causes an impairment of synaptic signalling in the developing and adult brain leading to learning and cognitive dysfunctions, it is therefore tempting to hypothesise that the reduction of electrochemical signalling from neurons to the tumor may inhibit the growth of gliomas in FXS [42]. Interestingly, recent findings shown that high-grade gliomas form aberrant synapses with healthy neurons that transmit electrical signals to the cancerous tissue. Neuronal activity through neuron-to-glioma synapses and the release of growth factors are emerging as crucial regulators of tumor progression [59]. The N-methyl-D-aspartate (NMDAR)-mediated signalling pathway appears to play an important role in GBM, increasing cell growth and invasiveness [80, 81]. Notably, FMRP levels are induced by NMDAR in a pancreatic neuroendocrine tumor [40]. Future studies are required to address if NMDAR signalling might also be involved in the upregulation of FMRP in GSCs.

In conclusion, deciphering the network of intricate interactions between RBPs and their cancer-related RNA targets is helping to contribute to our understanding of tumor biology, potentially unveiling new targets for therapy. Here, we highlight that in GSCs FMRP regulates a variety of different tumor mechanisms, such as proliferation and growth via the WNT signalling pathways. We found that the high FMRP levels observed in both GSCs and GBM tissues correlate with a worse patient outcome. Although further clinical studies are required, it is therefore tempting to envision in the future the use of FMRP as a potential biomarker for GBM, and FMRP may represent a potential molecular target for GBM therapies.

## Supplementary information

Supplementary infos

Original Data File

Supplementary Table 1

Supplementary Table 2

Supplementary Table 3

Supplementary Table 4

Supplementary Table 5

Reporting Summary

## Data Availability

All raw data supporting the findings of this study are available from the corresponding authors upon request.
