# Supplementary material for: FMRP modulates the Wnt signalling pathway in glioblastoma
Source: Cell Death Dis. 2022 Aug 18;13(8):719. doi: 10.1038/s41419-022-05019-w (PMC9388540; doi:10.1038/s41419-022-05019-w)
Supplement: Supplementary file 1 — Supplementary infos [file 41419_2022_5019_MOESM1_ESM.pdf]

Supplementary information

FMRP modulates the Wnt signaling pathway in glioblastoma

Giorgia Pedini, Mariachiara Buccarelli, Fabrizio Bianchi, Laura Pacini, Giulia Cencelli, Giorgio Q. D'Alessandris, Maurizio Martini, Stefano Giannetti, Franceschina Sasso, Valentina Melocchi, Maria Giulia Farace, Tilmann Achsel, Luigi M. Larocca, Lucia Ricci Vitiani, Roberto Pallini and Claudia Bagni

Supplementary Figures

Fig. S1 FMRP levels are upregulated in GSCs

Fig. S2 *FMR1* mRNA is actively translated in GSCs and its levels correlate with patient survival

Fig. S3 Reduction of FMRP and *FMR1* mRNA levels in three stably transduced GSCs

Fig. S4 Brain xenografts of human GFP-expressing GSC#148 in NOD-SCID mice

Fig. S5 FMRP levels significantly impairs cell growth of GSCs

Fig. S6 WNT signalling in ASD and in GSCs silenced for *FMR1* mRNA

Supplementary Figures

Figure S1

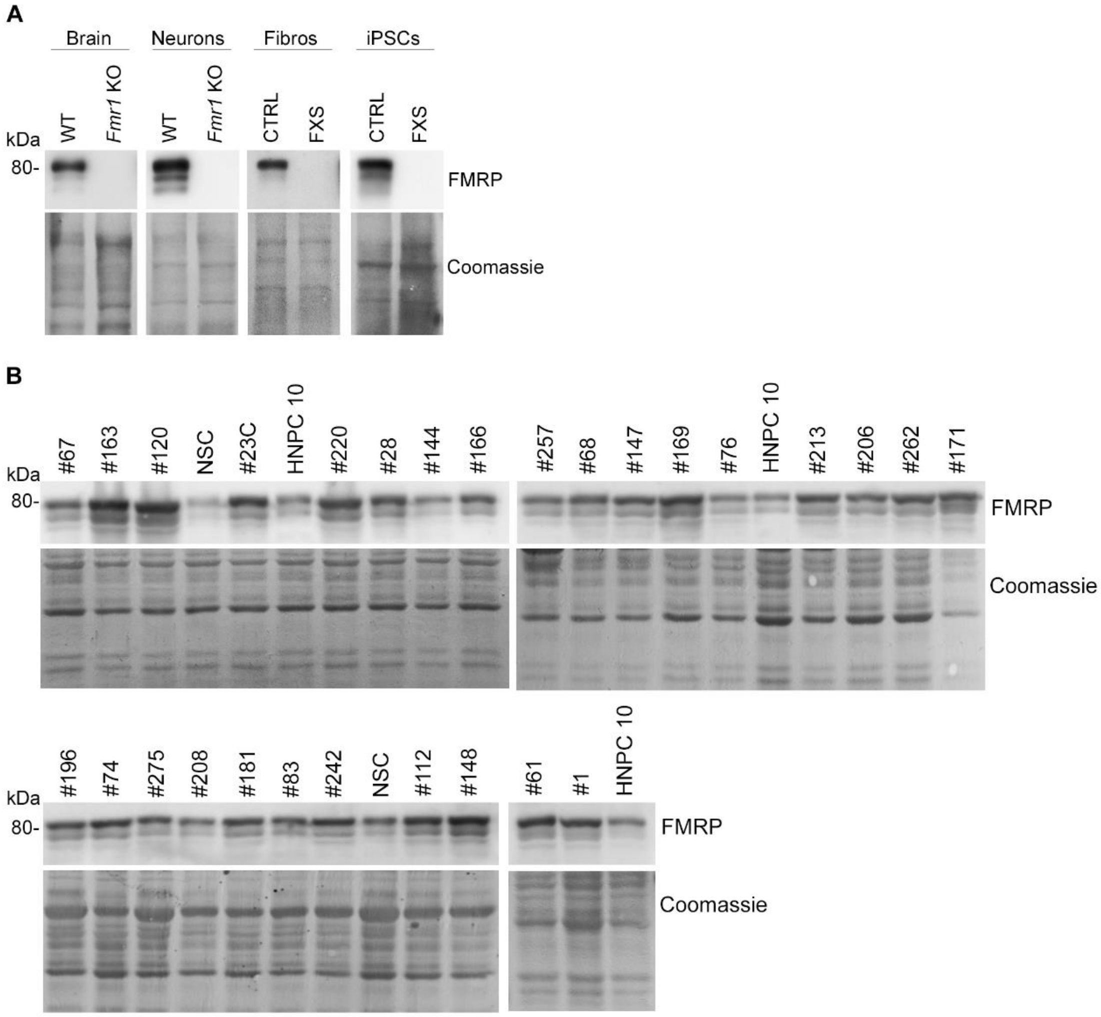

Fig. S1 FMRP levels are upregulated in GSCs. A FMRP was detected using PZ1 antibody in different protein extracts: mice brain and mice cortical neurons, from WT and *Fmr1* KO; human fibroblasts and human pluripotent stem cells (iPSCs), derived from healthy subjects (CTRL) and FXS individuals (FXS). **B** Representative western blot of FMRP levels in 28 different GSC lines and in human neural adult (NSC) and foetal (HNPC 10) control stem cells. Coomassie staining was used as normaliser. The samples were processed in parallel and replicates of western blots were performed.

Figure S2

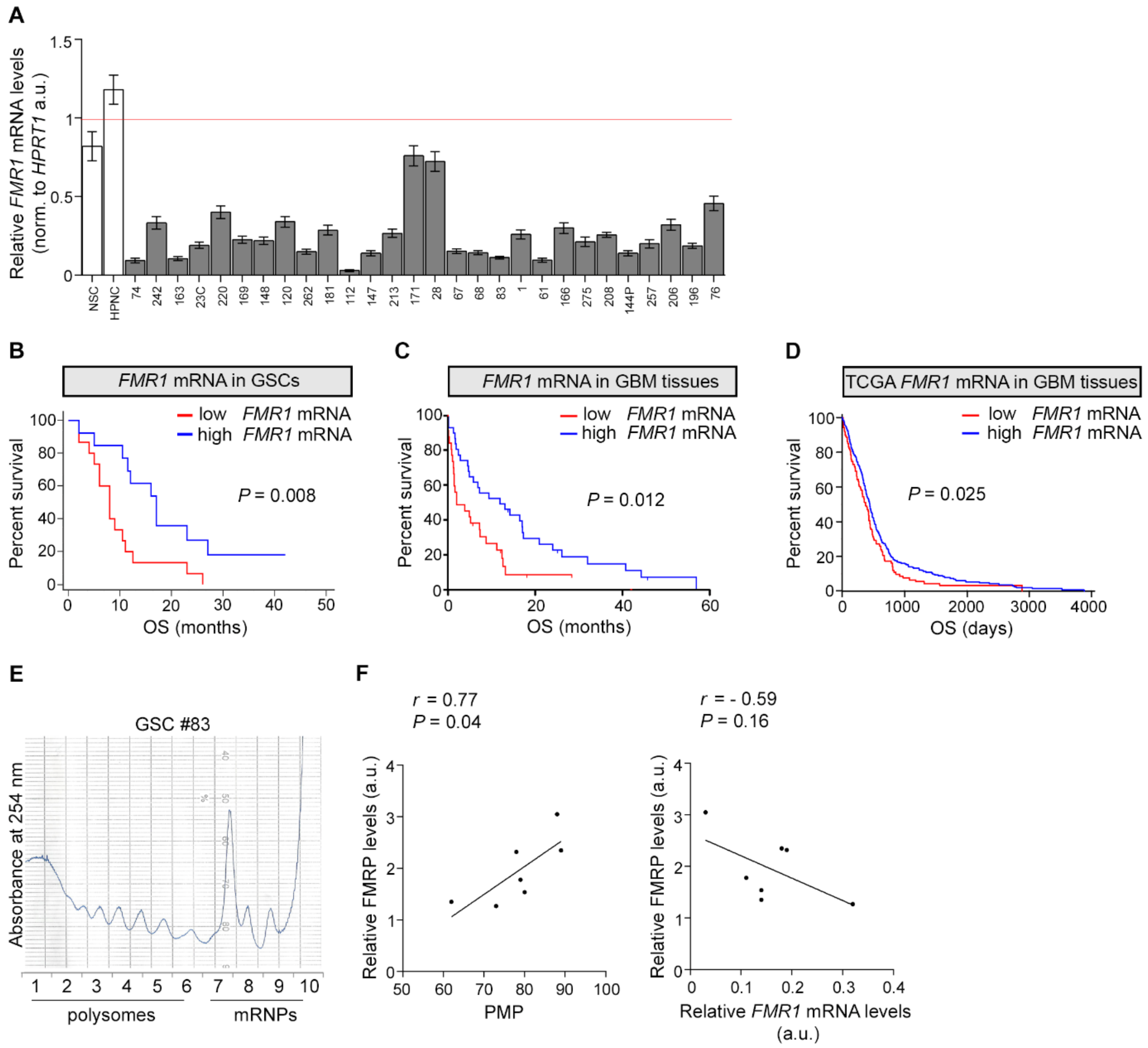

Fig. S2 *FMR1* mRNA is actively translated in GSCs and its levels correlate with patient survival. **A** Quantification of *FMR1* mRNA expression in 28 GSCs. Values were normalised for *HPRT1* mRNA levels and are shown relative to *FMR1* mRNA levels in NSC and fetal HPNC10 control cells, the average of which is set to 1. **B** Kaplan-Meier survival curve of GSCs showed that high *FMR1* mRNA expression (blue line; 12 GCS lines; median survival 17 months) was significantly associated to a better OS (*P* = 0.008; HR 0.30, 95% CI from 0.12 to 0.73) respect to low *FMR1* mRNA levels (red line; 16 GSC lines; median survival 8 months). **C** Kaplan-Meier survival curve of GBM patients stratified by *FMR1* mRNA expression. High *FMR1* mRNA expression (blue line; 30 cases; median survival 19 months) was significantly associated to a better OS (*P* = 0.012; HR 0.47, 95% CI from 0.26 to 0.84) respect to low *FMR1* mRNA levels (red line; 30 cases; median survival 5 months). **D** Kaplan-Meier survival curves of 523 GBM patients with different *FMR1* mRNA expression in the TCGA glioblastoma database, using the UCSC Xena software (https://xena.ucsc.edu/). GBM patients were grouped into low and high *FMR1* mRNA expression (based on the median expression value of *FMR1* mRNA). Lower levels of *FMR1* mRNA (red line; 107 cases; median survival 378 days) confer an unfavorable survival compared to high levels (blue line; 416 cases; median survival 440 days) (*P* = 0.025). **E** Representative UV absorbance profile of Polysomes/mRNPs cosedimentation analysis; absorbance at 254 nm is indicative of

rRNA concentrations. **F** Left panel, correlation between FMRP and *FMR1* mRNA levels and, right panel, FMRP levels and the percentage of *FMR1* mRNA on polysomes (PMP) in GSCs (in the graphs, the sample correlation coefficient *r* and the significance *P* in a Pearson correlation test are indicated).

Figure S3

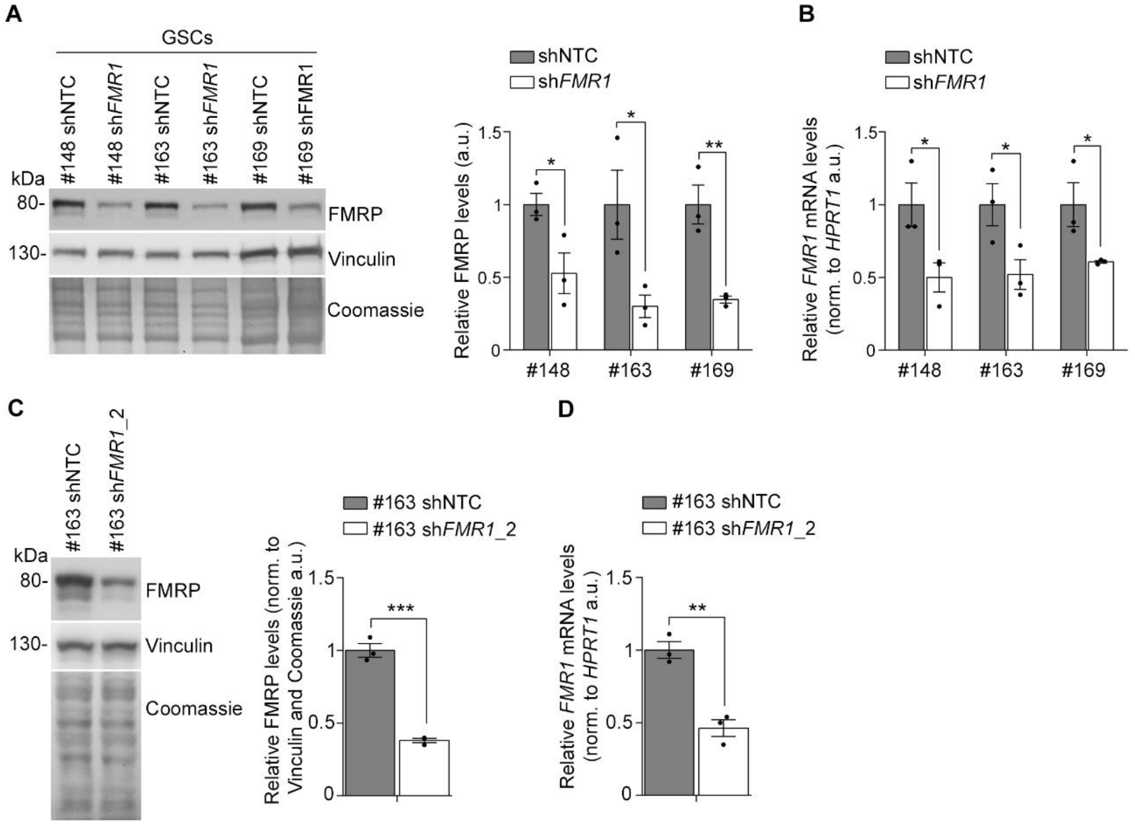

Fig. S3 Reduction of FMRP and *FMR1* mRNA levels in three stably transduced GSCs. A Left panel, representative western blot of FMRP protein levels in GSCs stable infected with the lentiviral empty vector (#148 shNTC, #163 shNTC and #169 shNTC) or with the lentiviral vector containing sh*FMR1* (#148 sh*FMR1*, #163 sh*FMR1*, #169 sh*FMR1*). Right panel, FMRP quantification in GSCs. Coomassie and Vinculin were used as normalisers (mean ± SEM, *n* = 3, *$P < 0.05$, **$P < 0.01$, Student's *t*-test). **B** RT-qPCR analysis of *FMR1* mRNA expression in GSCs infected with the empty vector (#148 shNTC, #163 shNTC and #169 shNTC) and with the vector containing sh*FMR1* (#148 sh*FMR1*, #163 sh*FMR1*, #169 sh*FMR1*). *HPRT1* mRNA was used as normaliser (mean ± SEM, *n* = 3, *$P < 0.05$, Student's *t*-test). **C** Left panel, representative western blot of FMRP protein levels in GSCs transduced with the lentiviral empty vector (#163 shNTC) or with the second lentiviral vector sh*FMR1* (#163 sh*FMR1*_2). Right panel, FMRP quantification in GSCs. Vinculin and Coomassie staining were used as normalisers. **D** RT-qPCR analysis of *FMR1* mRNA expression in GSCs transduced with the empty vector (#163 shNTC) and with the second lentiviral vector sh*FMR1* (#163 sh*FMR1*_2). *HPRT1* mRNA was used as normaliser (mean ± SEM, *n* = 3, **$P < 0.01$, ***$P < 0.001$, Student's *t*-test).

Figure S4

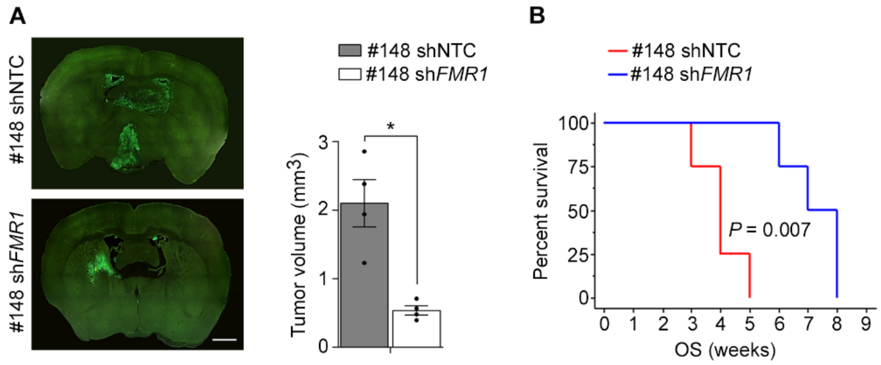

Fig. S4 Brain xenografts of human GFP-expressing GSC#148 in NOD-SCID mice. **A** Left panel, coronal brain sections showing tumor growth into and around the ventricles of shNTC GSC#148 (control) xenograft. Brain xenografts of sh*FMR1* GSC#148 showed remarkable reduction of tumor growth. Right panel, quantification of tumor volumes (mean ± SEM, $n = 4$, *$P < 0.05$, Student's t-test). Scale bar, 800 µm. **B** Kaplan-Meier curve estimating the survival of mice grafted with shNTC GSC#148 (red line; 4 mice; median survival 4 weeks) and sh*FMR1* GSC#148 (blue line; 4 mice; median survival 7 weeks) ($P = 0.007$, log-rank test).

Figure S5

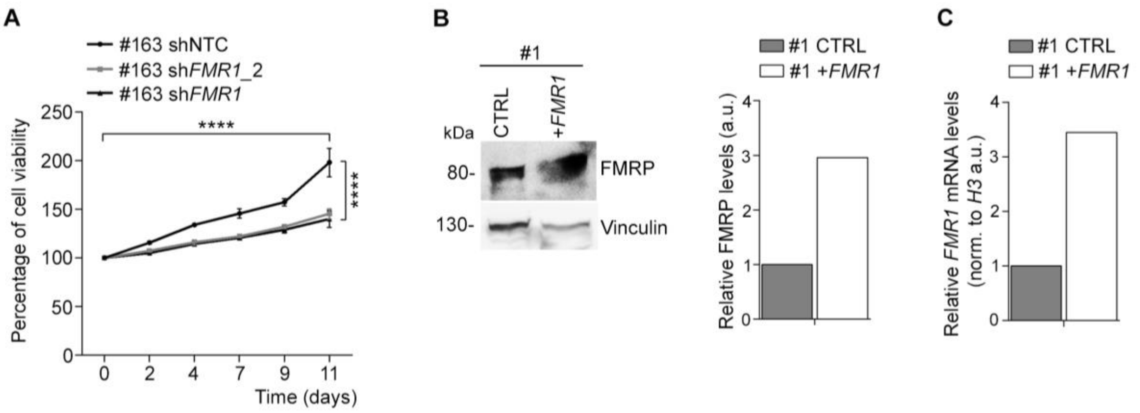

Fig. S5 FMRP levels significantly impair cell growth of GSCs. **A** Growth curves of GSCs transduced with either shNTC or two different sh*FMR1* vectors (as indicated on top of each plot). Days and percentage of cell number are shown (mean ± SEM, n = 3, ****P < 0.0001, two-way ANOVA). **B** Left panel, representative Western blot of FMRP protein levels in GSC#1 transduced with the empty vector (CTRL) or with the vector containing overexpressed FMRP (+FMRP). Right panel, FMRP quantification in GSC#1. Vinculin was used as normaliser. **C** RT-qPCR analysis of *FMR1* mRNA expression in GSC#1 transduced with the empty vector (CTRL) or with the vector containing overexpressed FMRP (+FMRP). *H3* mRNA was used as normaliser.

Figure S6

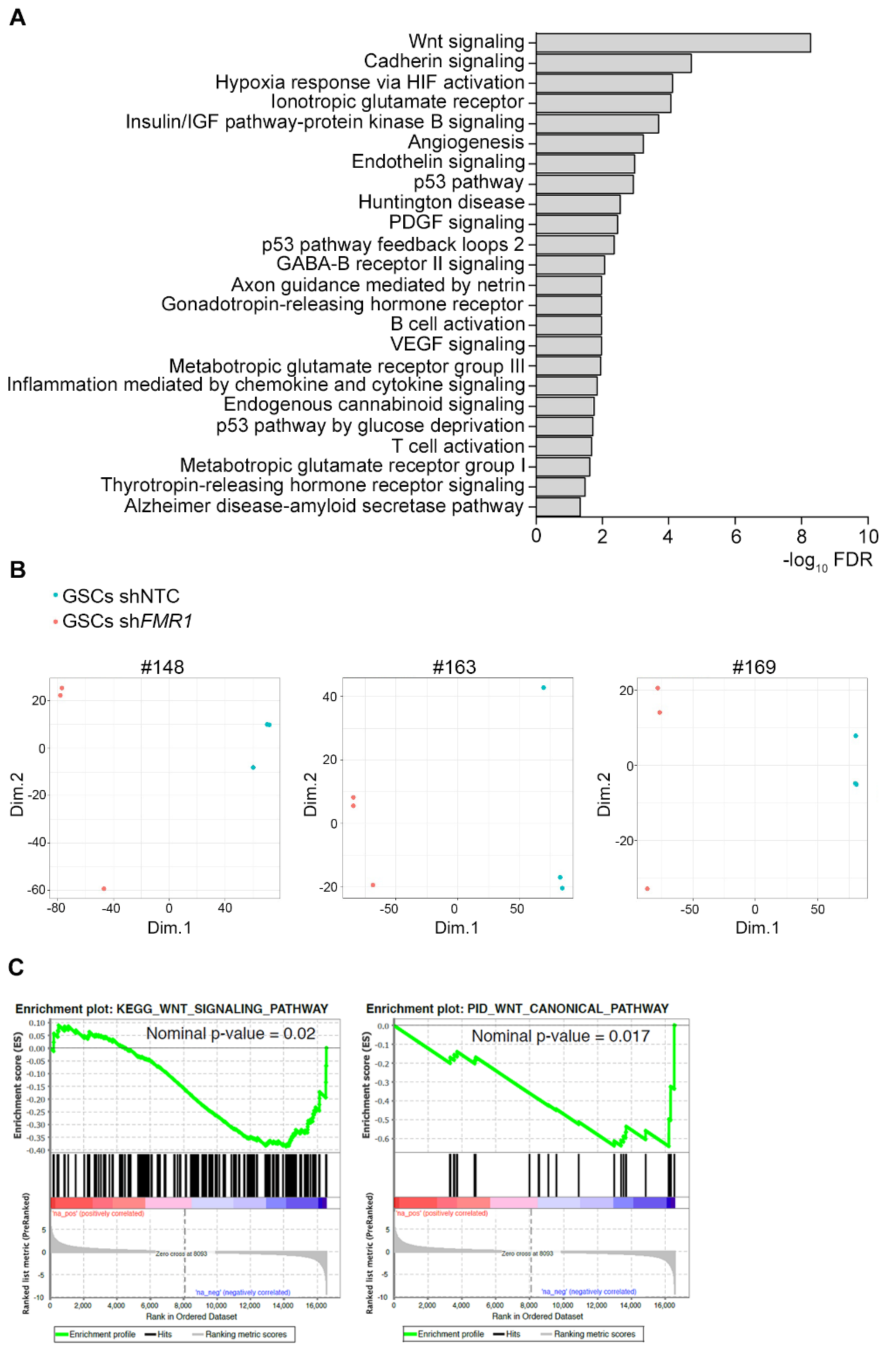

Fig. S6 WNT signaling in ASD and in GSCs silenced for *FMR1* mRNA. **A** Enrichment analysis of genes that are found in the SFARI database and are mutated in GBM according to the Cancer Genome Atlas. Shown are Panther pathways that are significantly enriched (FDR < 0.05). **B** Transcriptome clustering based on unweighted Principal Component Analysis (PCA) of RNAs derived from shNTC (orange dots) and sh*FMR1* (light blue dots) GSCs (n = 3/genotype/group). Of note, the shNTC and sh*FMR1* treated samples are widely separated along the first PCA dimension (Dim.1) representing most of the variance in the datasets. **C** Representative GSEA plots showing significant (P < 0.05) WNT-related gene sets negatively enriched in sh*FMR1* GSCs. NES, normalized enrichment score. Nominal p-value as well as false discovery rate (FDR) are shown.
