# Supplementary material for: FMRP modulates the Wnt signalling pathway in glioblastoma
Source: Cell Death Dis. 2022 Aug 18;13(8):719. doi: 10.1038/s41419-022-05019-w (PMC9388540; doi:10.1038/s41419-022-05019-w)

Original Western Blots

FMRP modulates the Wnt signaling pathway in glioblastoma

Giorgia Pedini, Mariachiara Buccarelli, Fabrizio Bianchi, Laura Pacini, Giulia Cencelli, Quintino G. D'Alessandris, Maurizio Martini, Stefano Giannetti, Franceschina Sasso, Valentina Melocchi, Maria Giulia Farace, Tilmann Achsel, Luigi M. Larocca, Lucia Ricci Vitiani, Roberto Pallini and Claudia Bagni

Figure 4_Original western blots

C

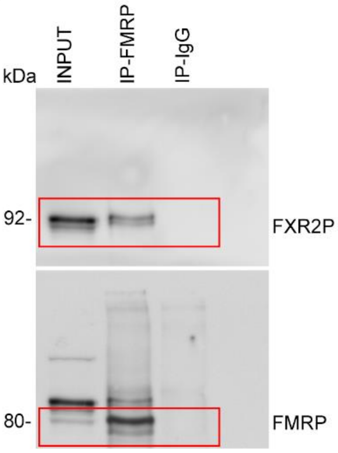

Figure 5_Original western blots

A

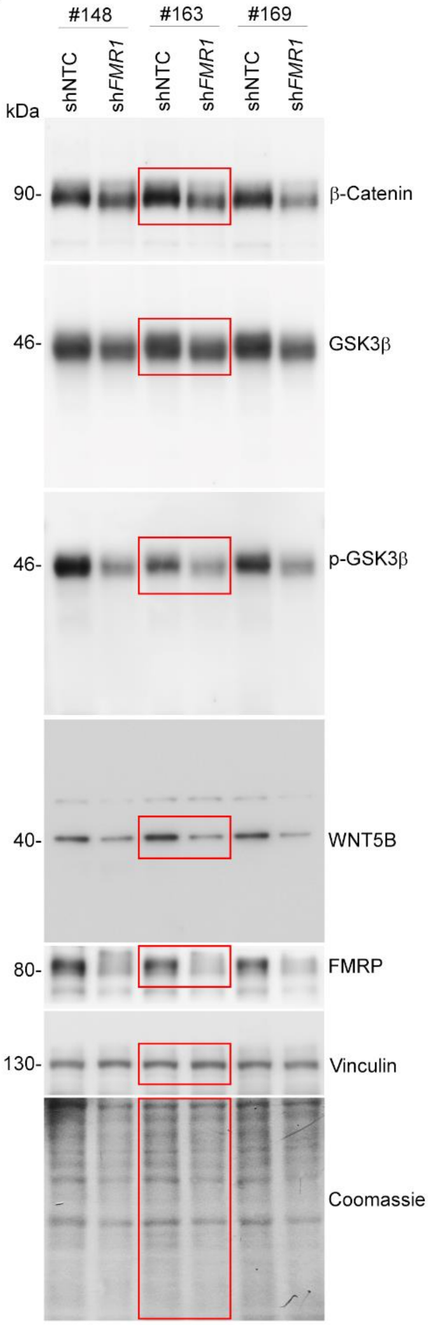

Figure 5_Original western blots

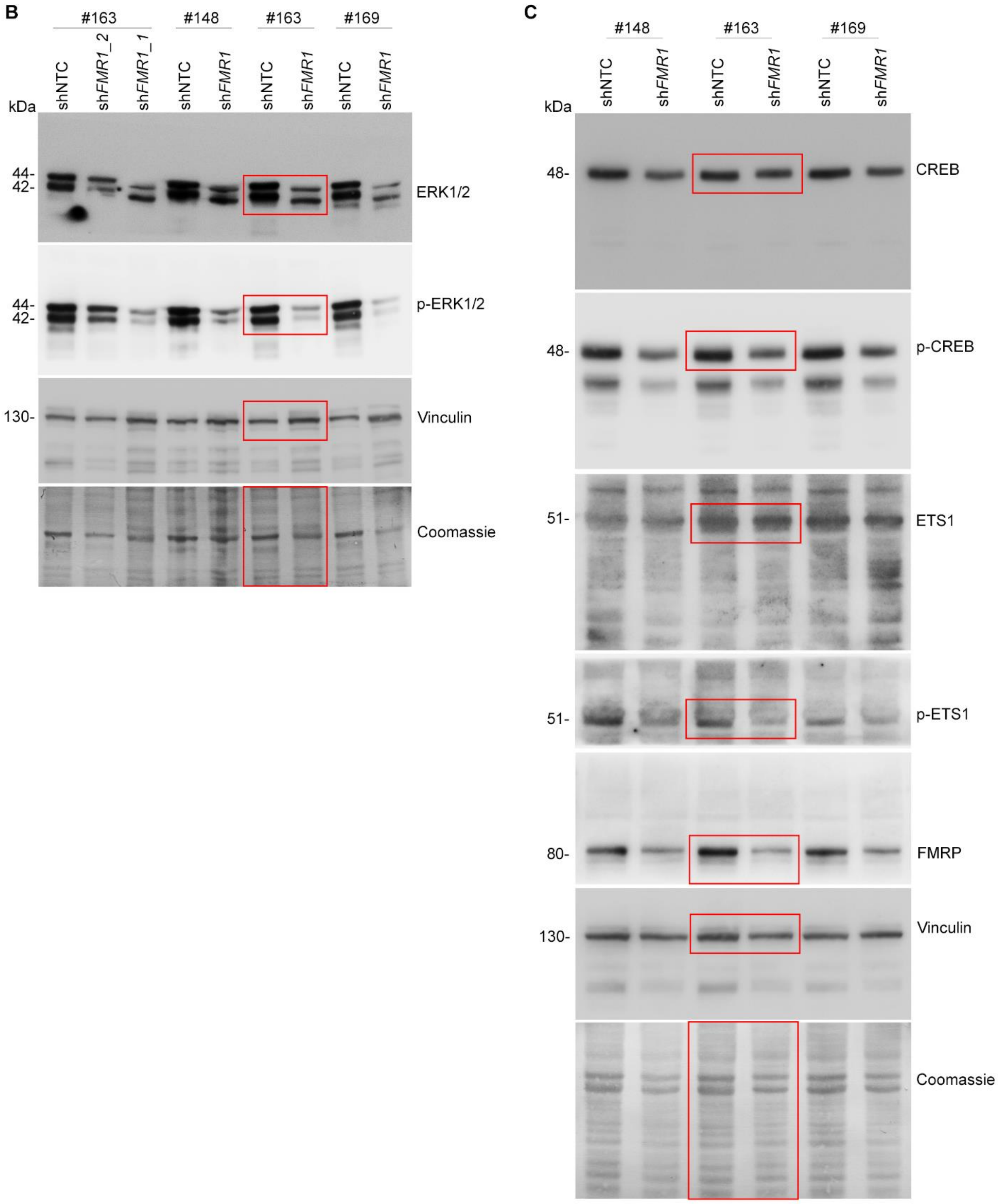

Figure S1_Original western blots

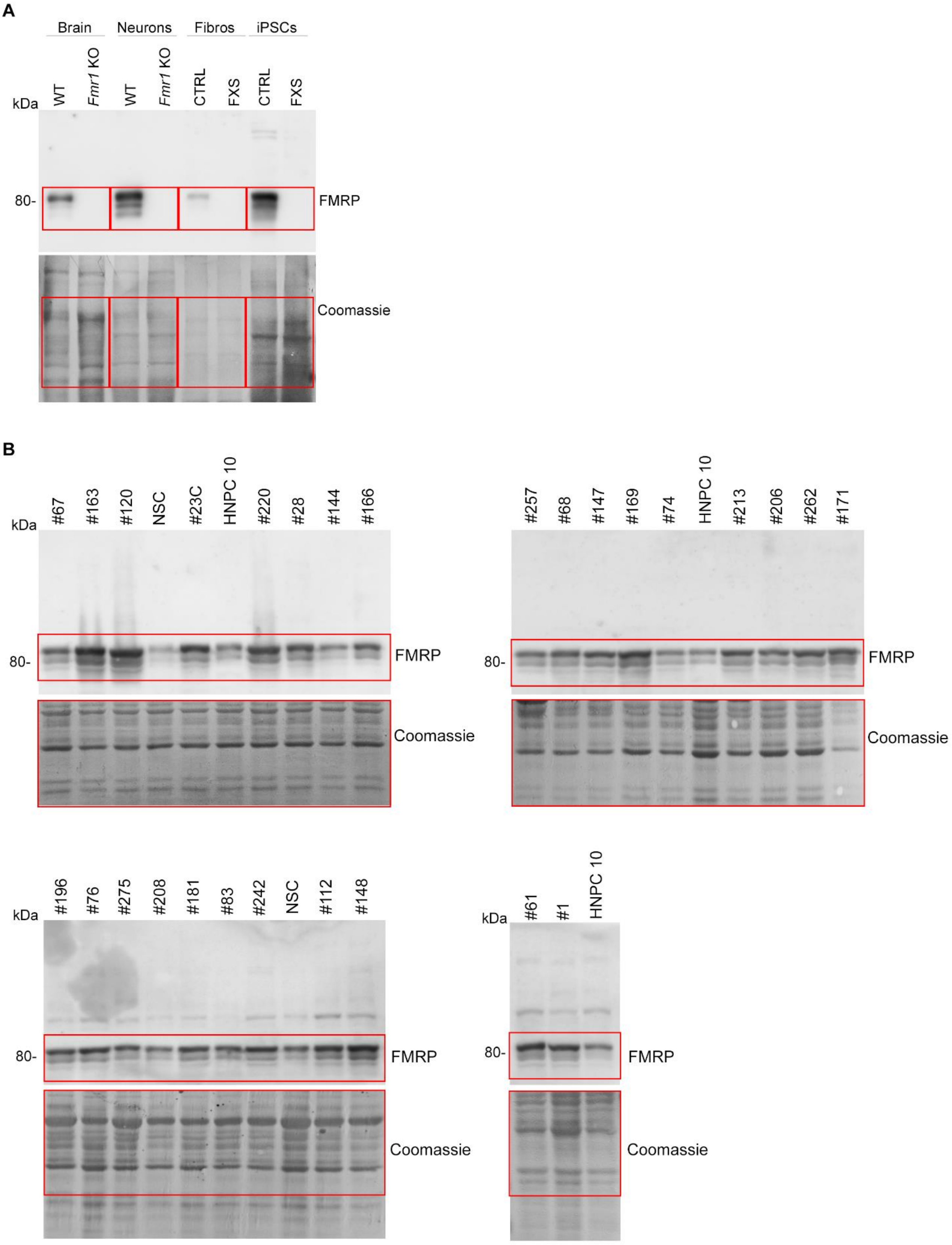

Figure S3_Original western blots

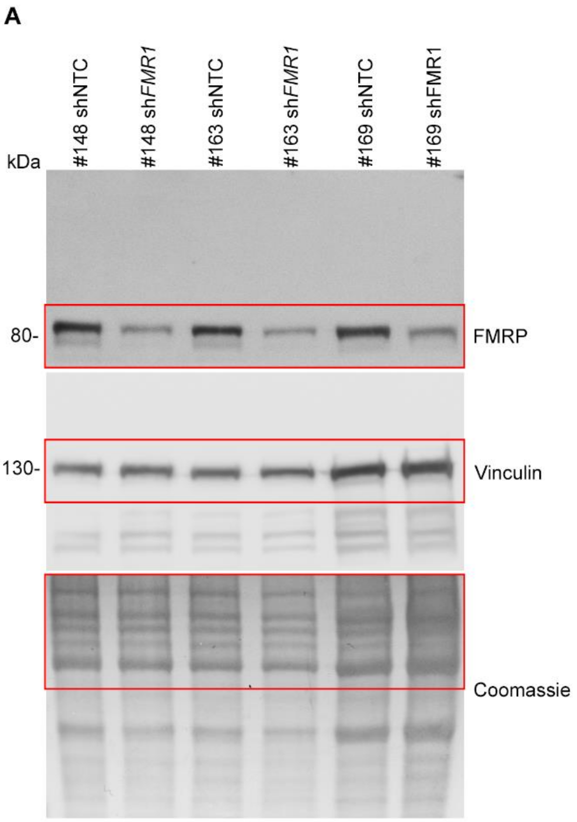

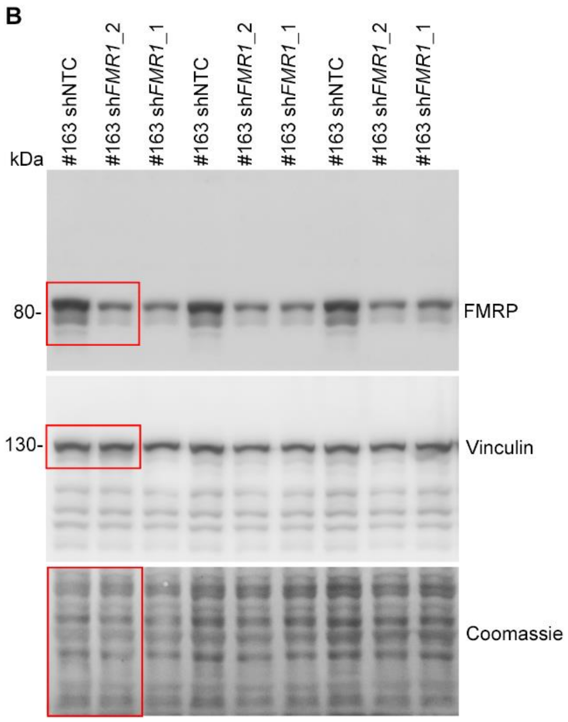

Figure 5_Original western blots

B

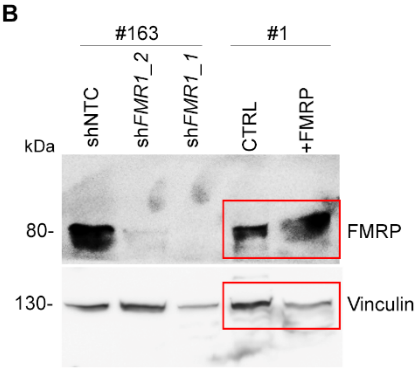

Supplement: Supplementary file 2 — Original Data File [file 41419_2022_5019_MOESM2_ESM.pdf]
